# Supplementary material for: A Genome-Wide Analysis of Nuclear Mitochondrial DNA Sequences (NUMTs) in Chrysomelidae Species (Coleoptera)
Source: Insects. 2025 Feb 2;16(2):150. doi: 10.3390/insects16020150 (PMC11856540; doi:10.3390/insects16020150)
Supplement: Supplementary file 1 [file insects-16-00150-s001.zip › Figure-Supplementary Materials.pdf]

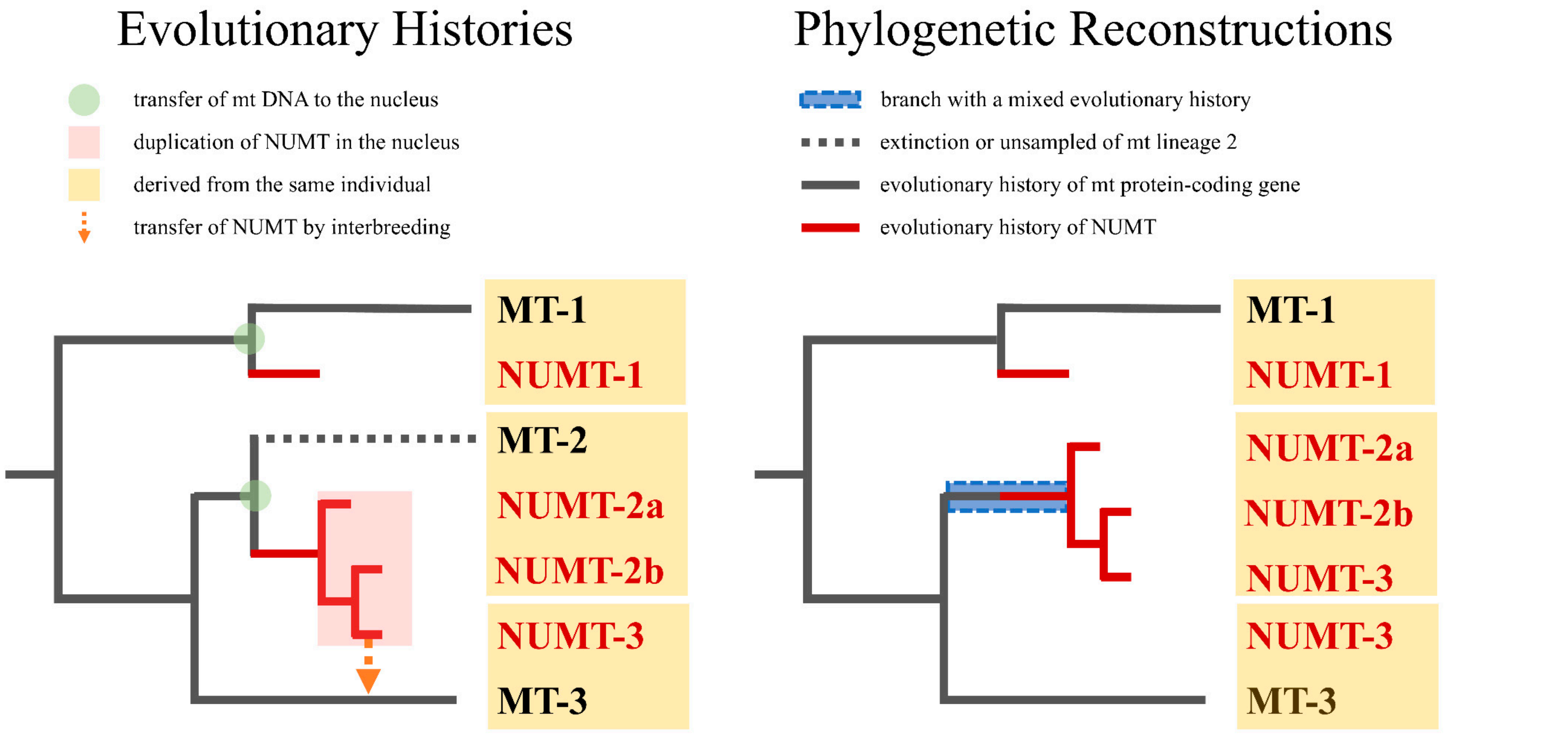

Figure S1: Dynamics of NUMTs generation and evolution along mitochondrial (mt) lineages (left) and the resulting phylogenetic reconstructions (right). Based on Baldo et al., with significant modifications by the authors. The third-codon-position bias should not be observed between the paired sequences of NUMT-2a, NUMT-2b, and NUMT-3, as well as between MT-1 and NUMT-1. However, the mutation pattern of the paired sequences from the same individual, MT-3 and NUMT-3, should exhibit the third-codon-position bias.

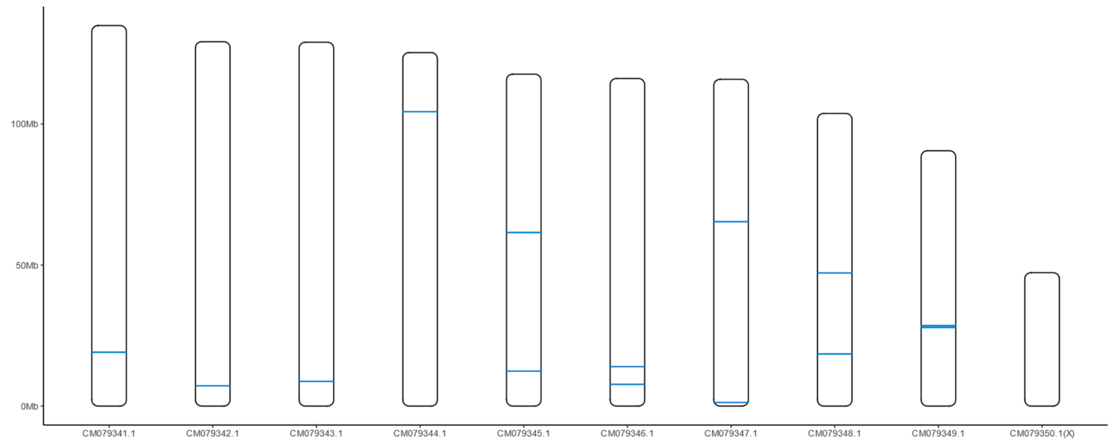

(a) *Callosobruchus maculatus*

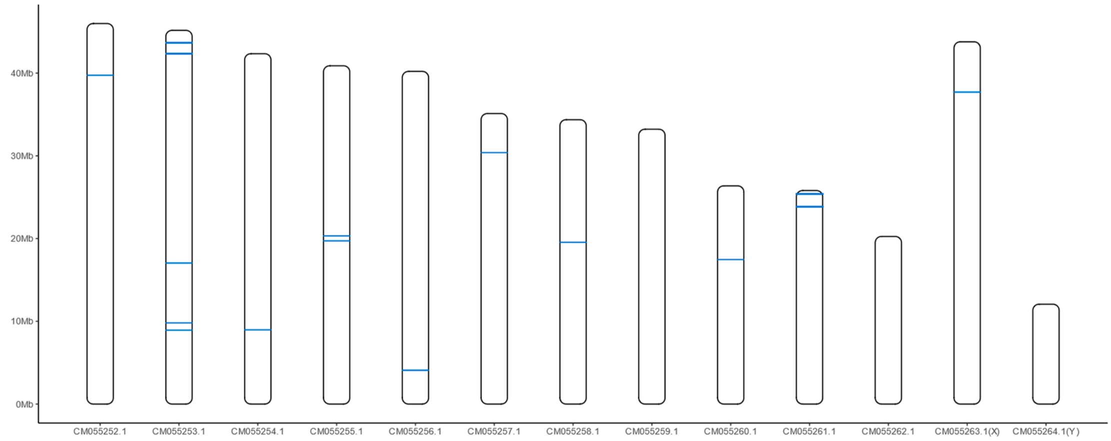

(b) *Diorhabda carinata*

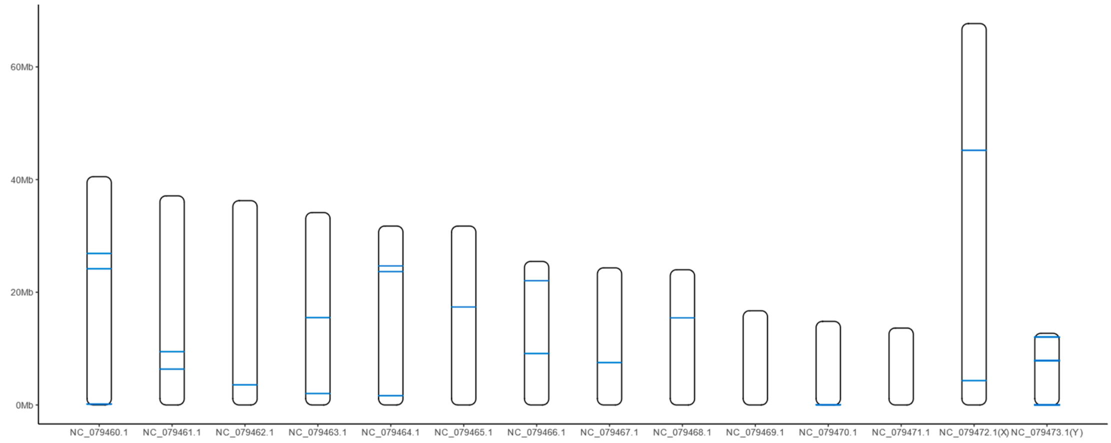

(c) *Diorhabda carinulata*

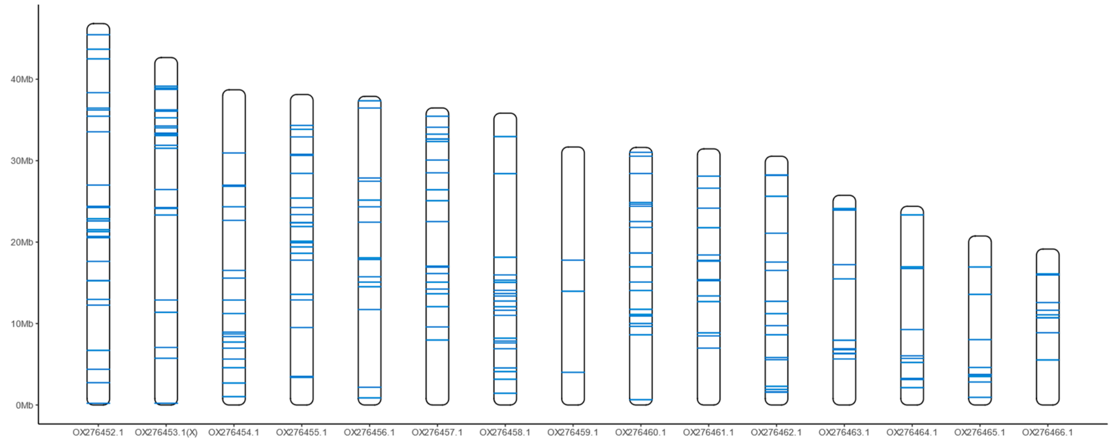

(d) *Cryptocephalus moraei*

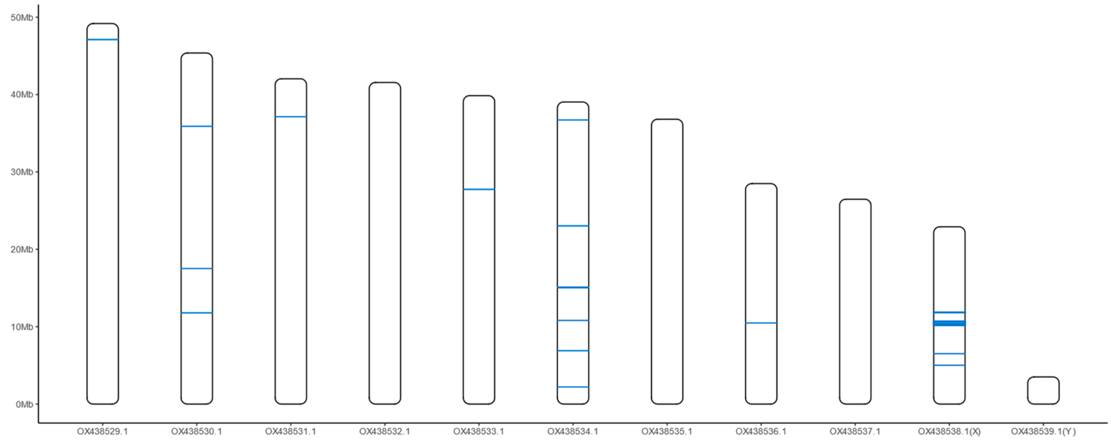

(e) *Bruchidius siliquastri*

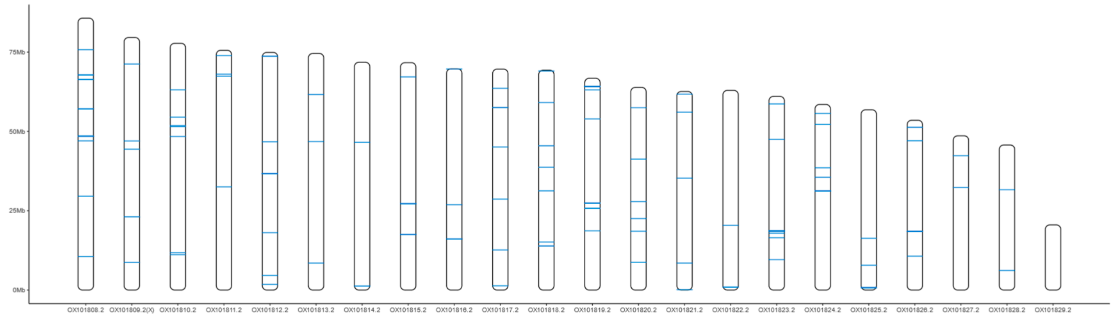

(f) *Chrysolina oricalcia*

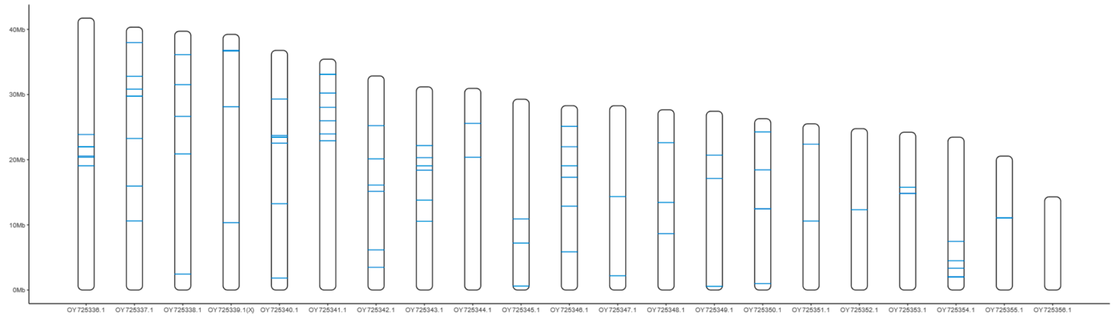

(g) *Neocrepidodera transversa*

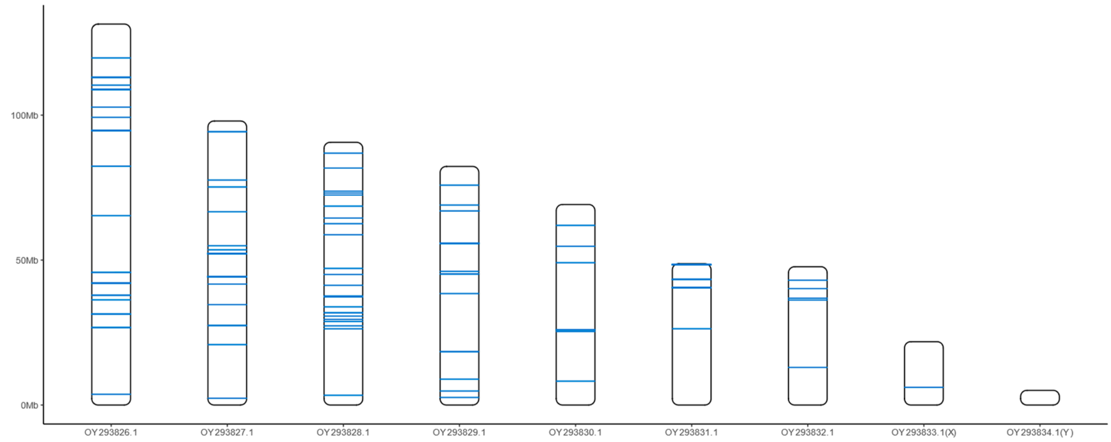

(h) *Crioceris asparagi*

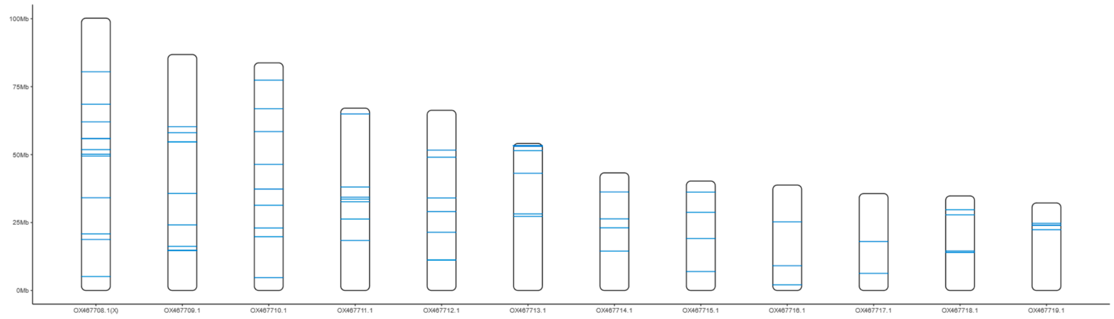

(i) *Agelastica alni*

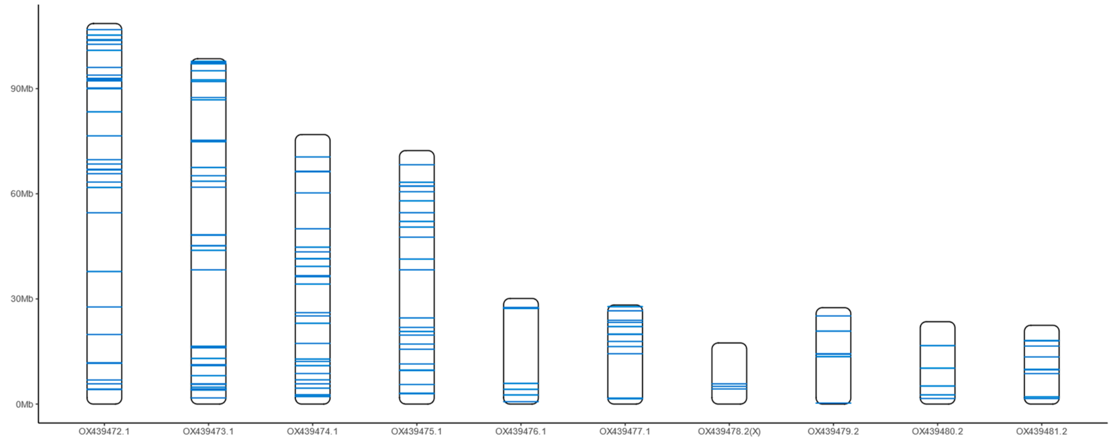

(j) *Crepidodera aurea*

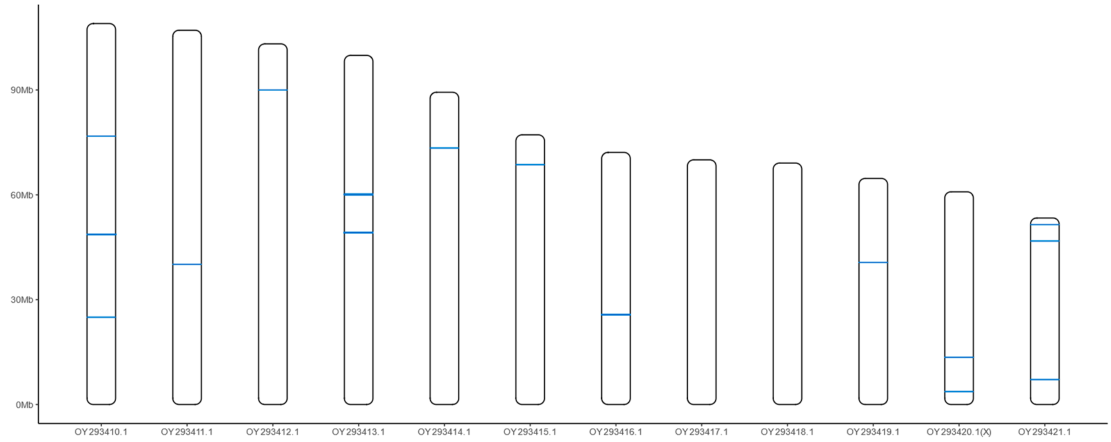

(k) *Chrysolina americana*

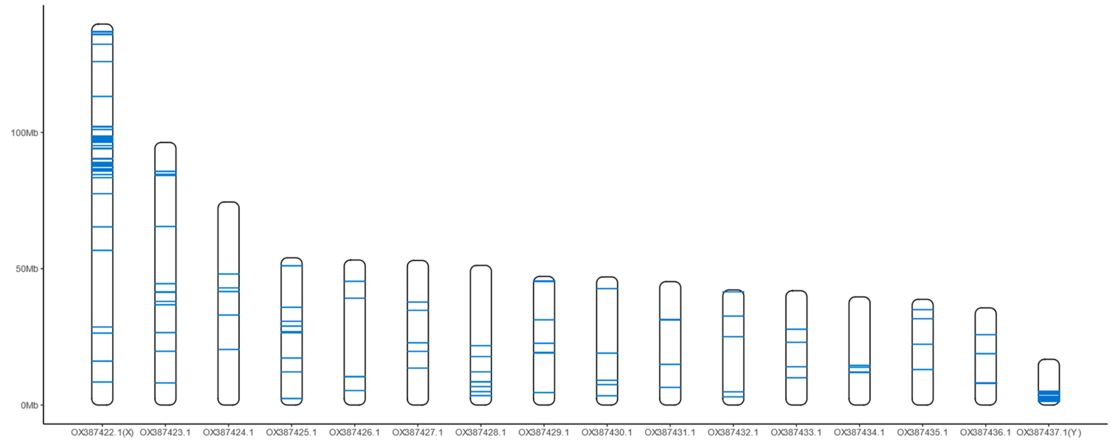

(l) *Lochmaea crataegi*

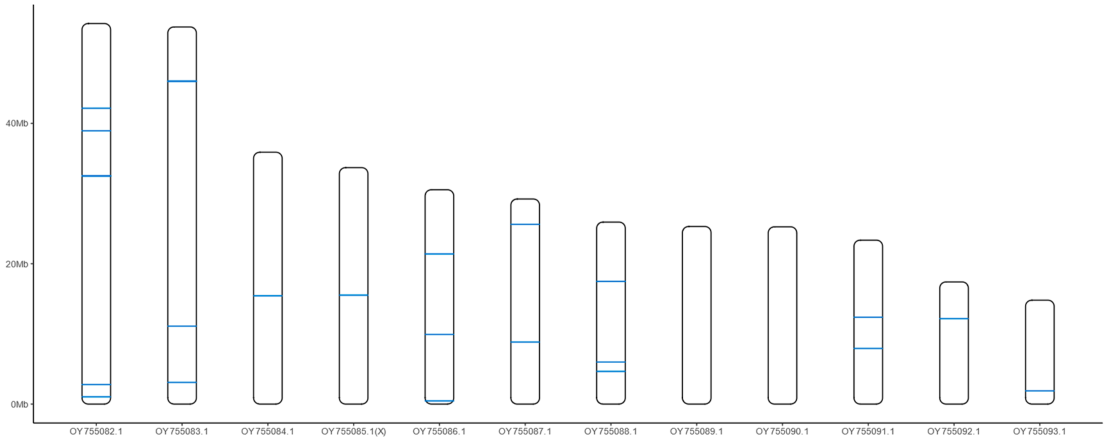

(m) *Gastrophysa polygoni*

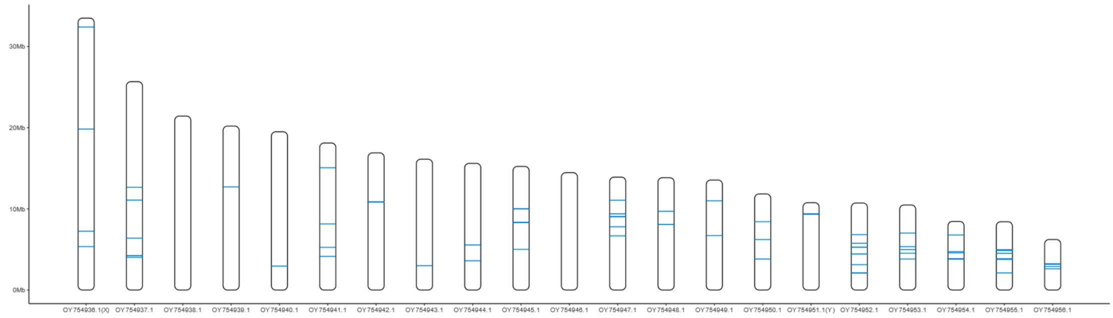

(n) *Cryptocephalus primarius*

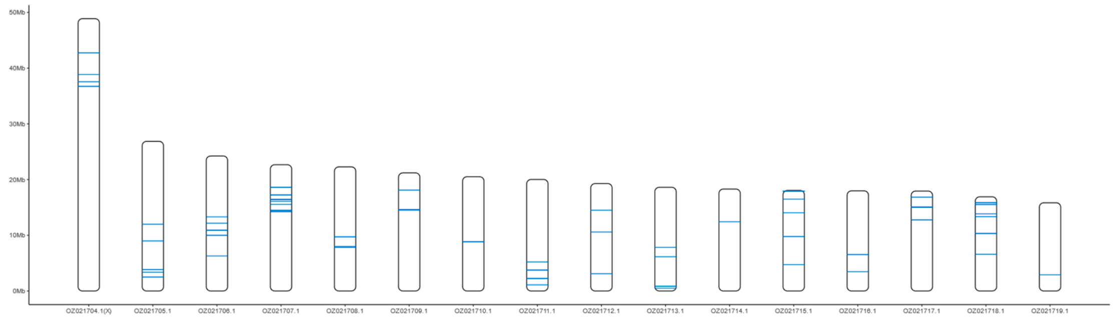

(o) *Galerucella nymphaeae*

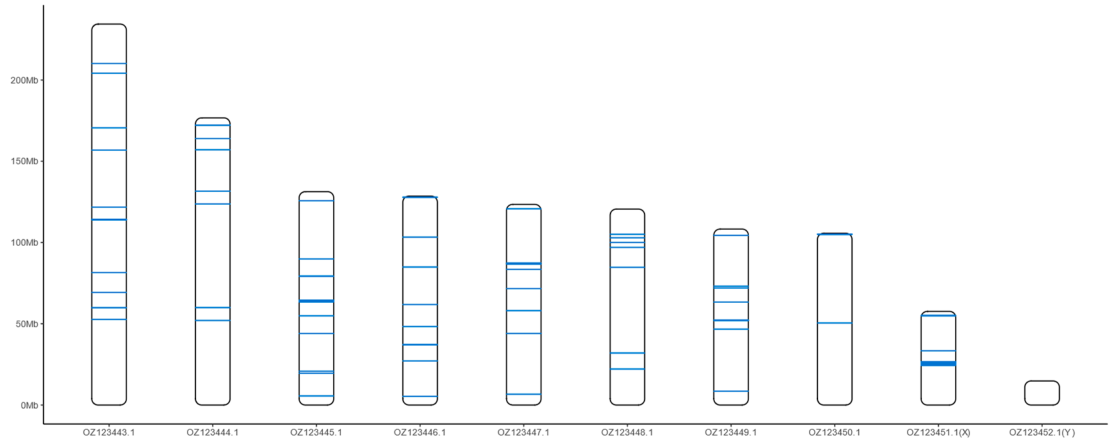

(p) *Bruchidius varius*

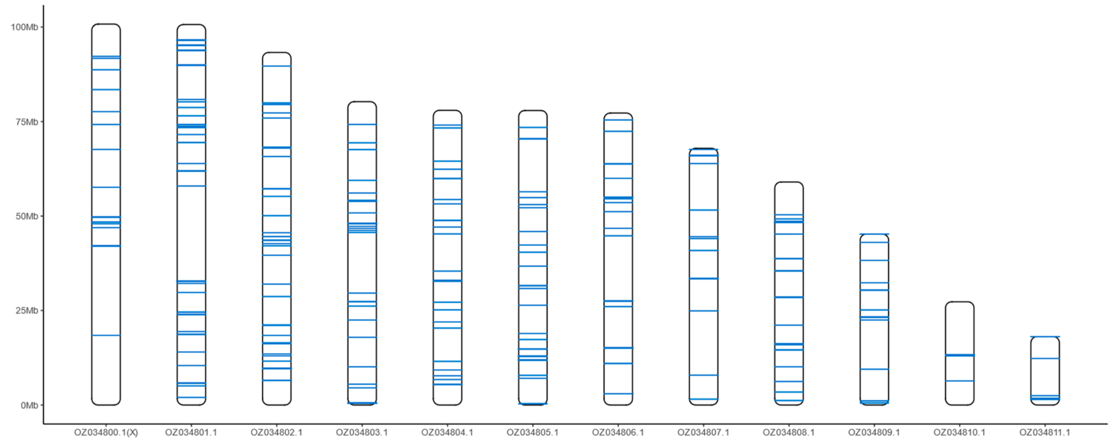

(q) *Altica lythri*

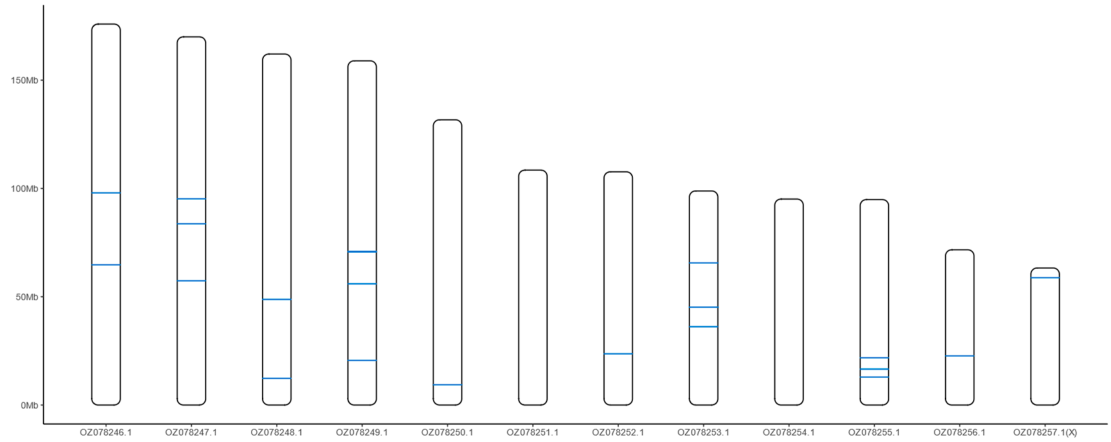

(r) *Chrysolina graminis*

Figure S2: Chromosomal localization of NUMTs derived from 13 mitochondrial PCGs in Chrysomelidae species with annotated sex chromosomes. (a) *Callosobruchus maculatus*; (b) *Diorhabda carinata*; (c) *Diorhabda carinulata*; (d) *Cryptocephalus moraei*; (e) *Bruchidius siliquastri*; (f) *Chrysolina oricalcia*; (g) *Neocrepidodera transversa*; (h) *Crioceris asparagi*; (i) *Agelastica alni*; (j) *Crepidodera aurea*; (k) *Chrysolina americana*; (l) *Lochmaea crataegi*; (m) *Gastrophysa polygoni*; (n) *Cryptocephalus primaries*; (o) *Galerucella nymphaeae*; (p) *Bruchidius varius*; (q) *Altica lythri*; (r) *Chrysolina graminis*.

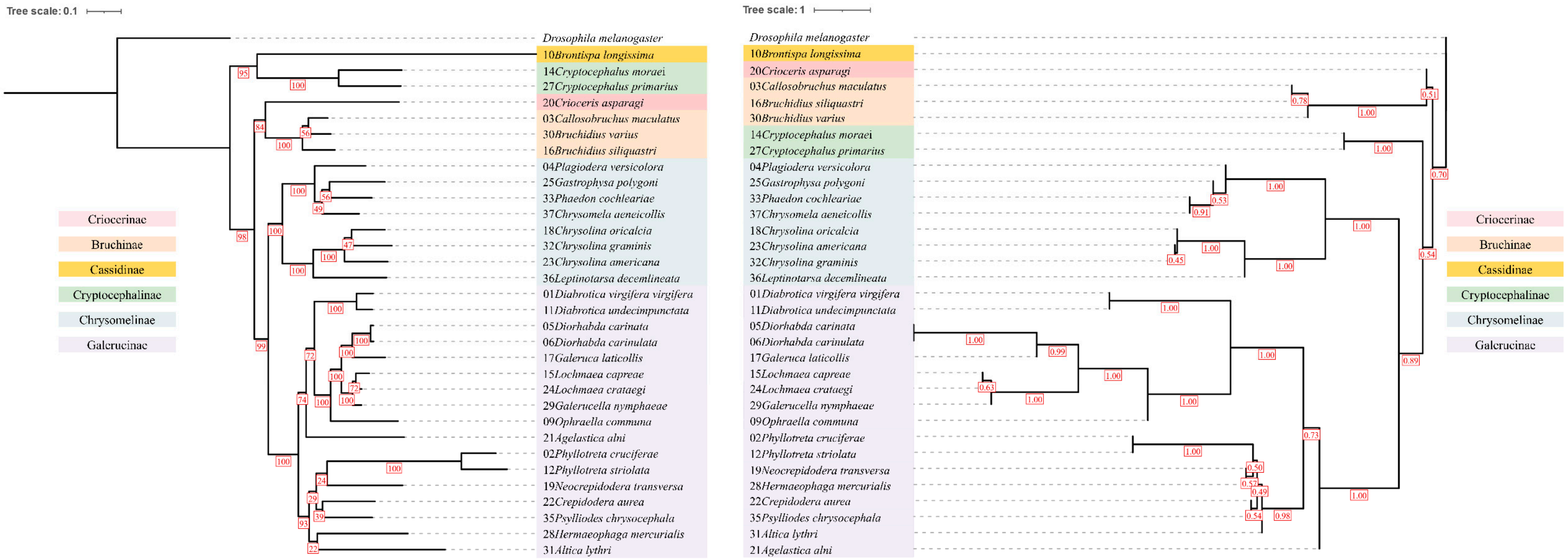

(a) **(b)**

Figure S3: ML tree based on the 13 PCGs amino acid sequence (13PCGs-AA) dataset. (**a**) Supermatrix concatenation method, with red-boxed numbers indicating node support as bootstrap values; (**b**) Multispecies coalescent method, with red-boxed numbers indicating node support as local posterior probabilities.

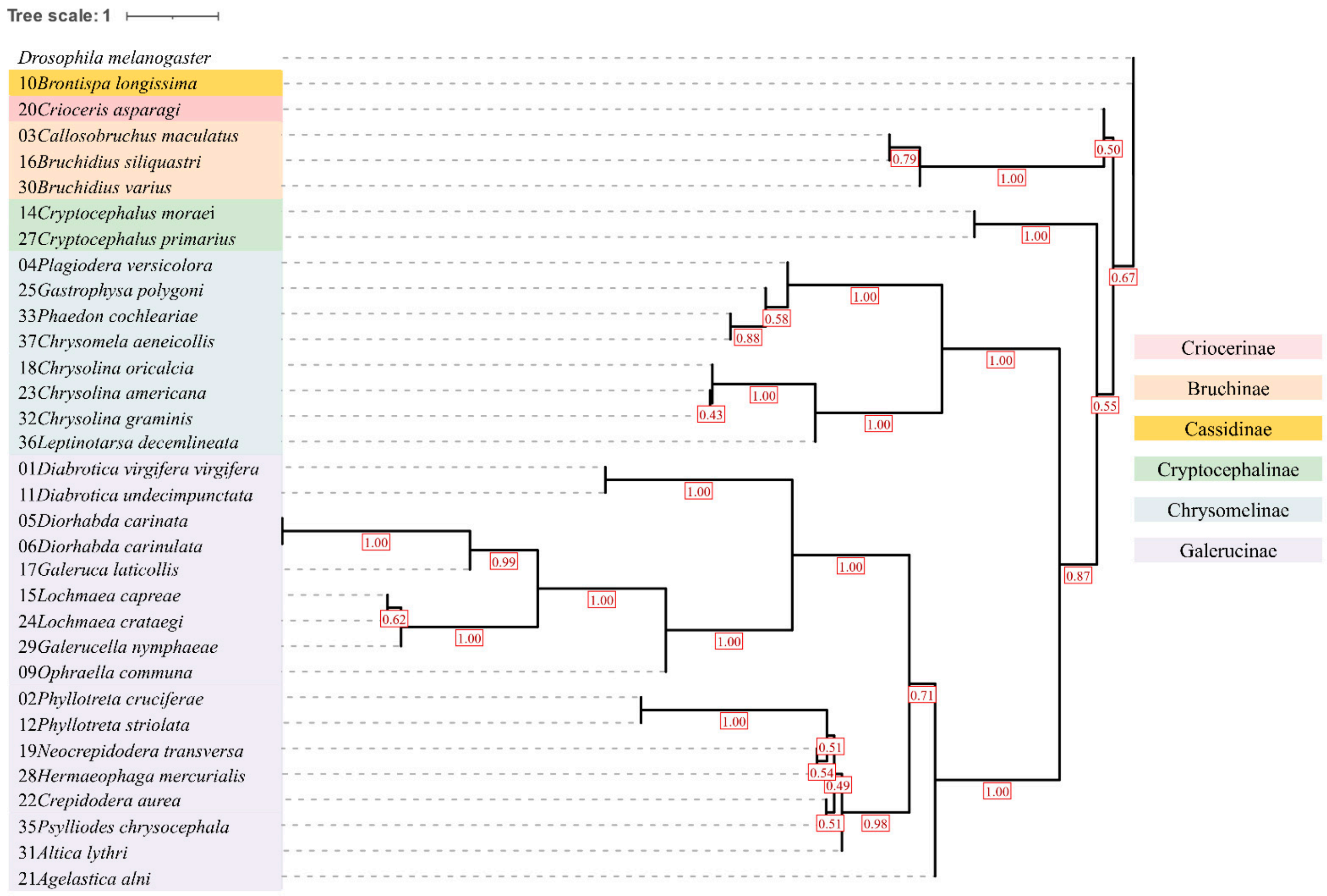

Figure S4: ML tree based on the 11PCGs-AA dataset using the multispecies coalescent method. The numbers in red boxes indicate node support as local posterior probabilities.

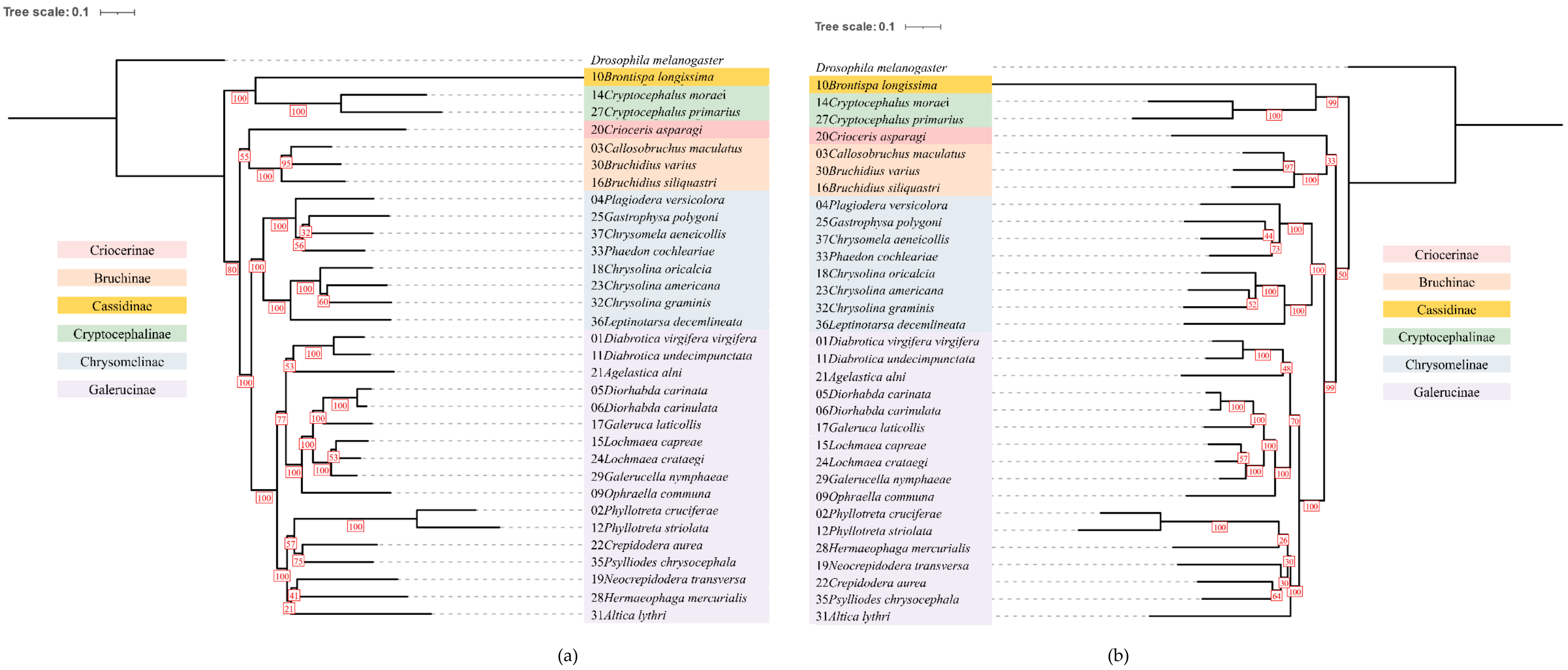

Figure S5: ML tree based on two nucleotide sequence datasets. (**a**) the 13PCGs-CDS dataset; (**b**) the 11PCGs-CDS dataset. The numbers in red boxes indicate node support as bootstrap values.

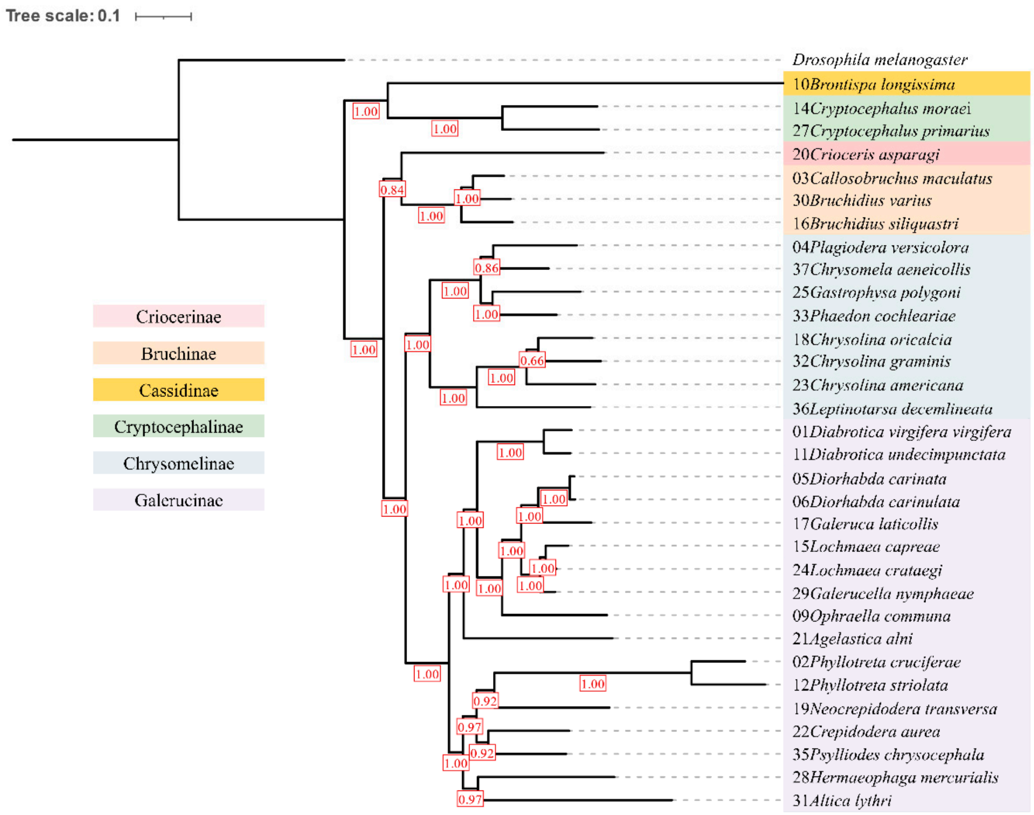

Figure S6: BI tree based on the 13PCGs-AA dataset. The numbers in red boxes indicate node support as posterior probabilities.

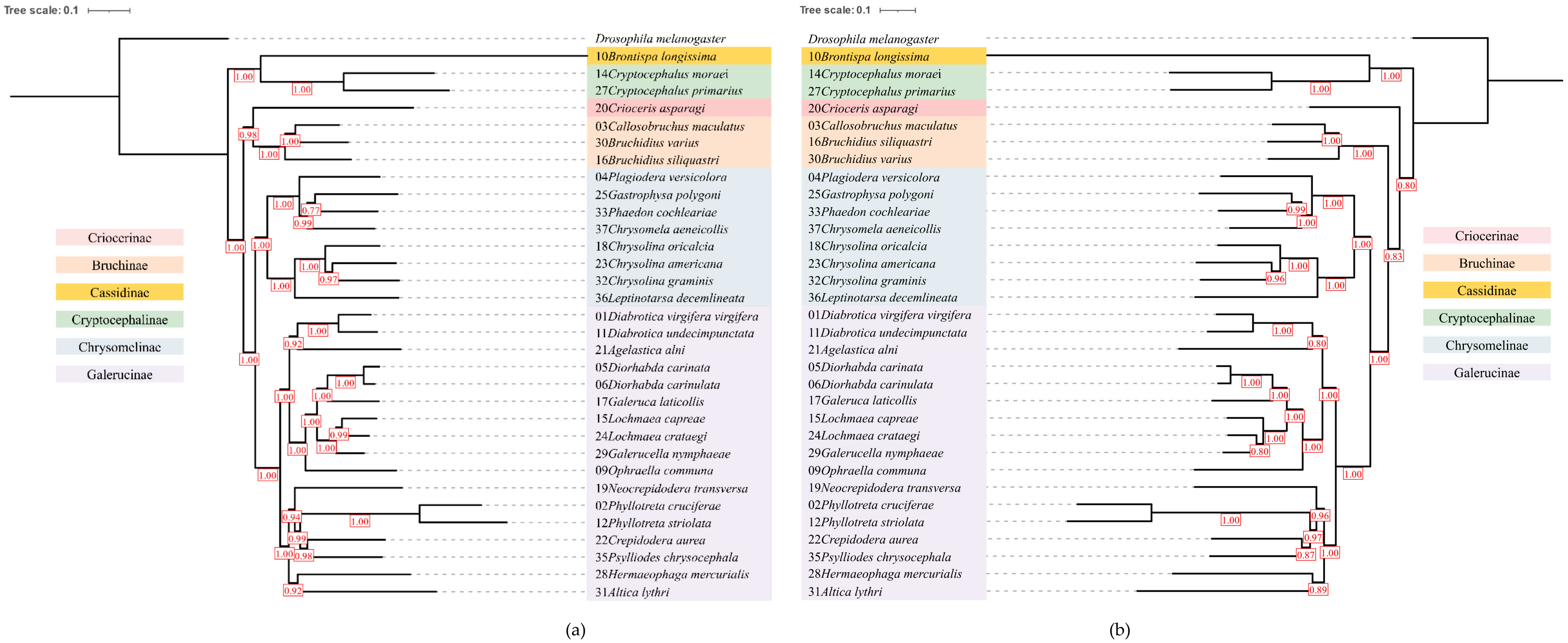

Figure S7: BI tree based on two nucleotide sequence datasets. (**a**) the 13PCGs-CDS dataset; (**b**) the 11PCGs-CDS dataset. The numbers in red boxes indicate node support as posterior probabilities.

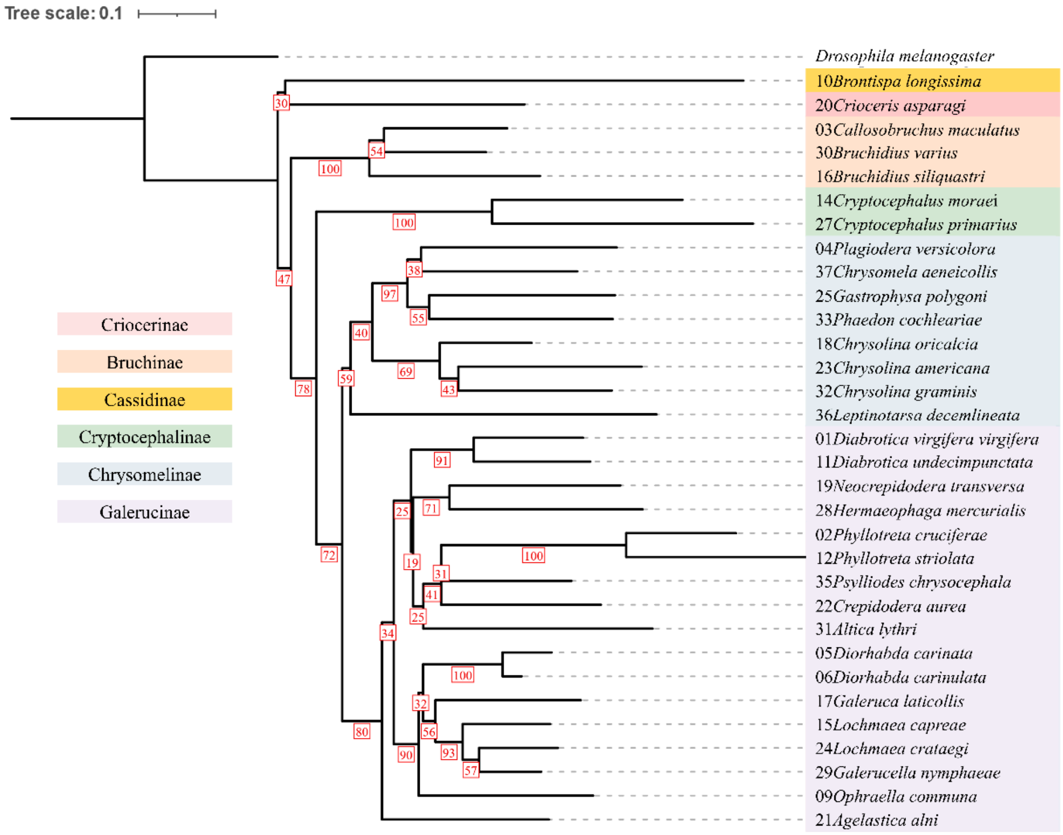

Figure S8: ML tree based on the *COX1* dataset. The numbers in red boxes indicate node support as bootstrap values.

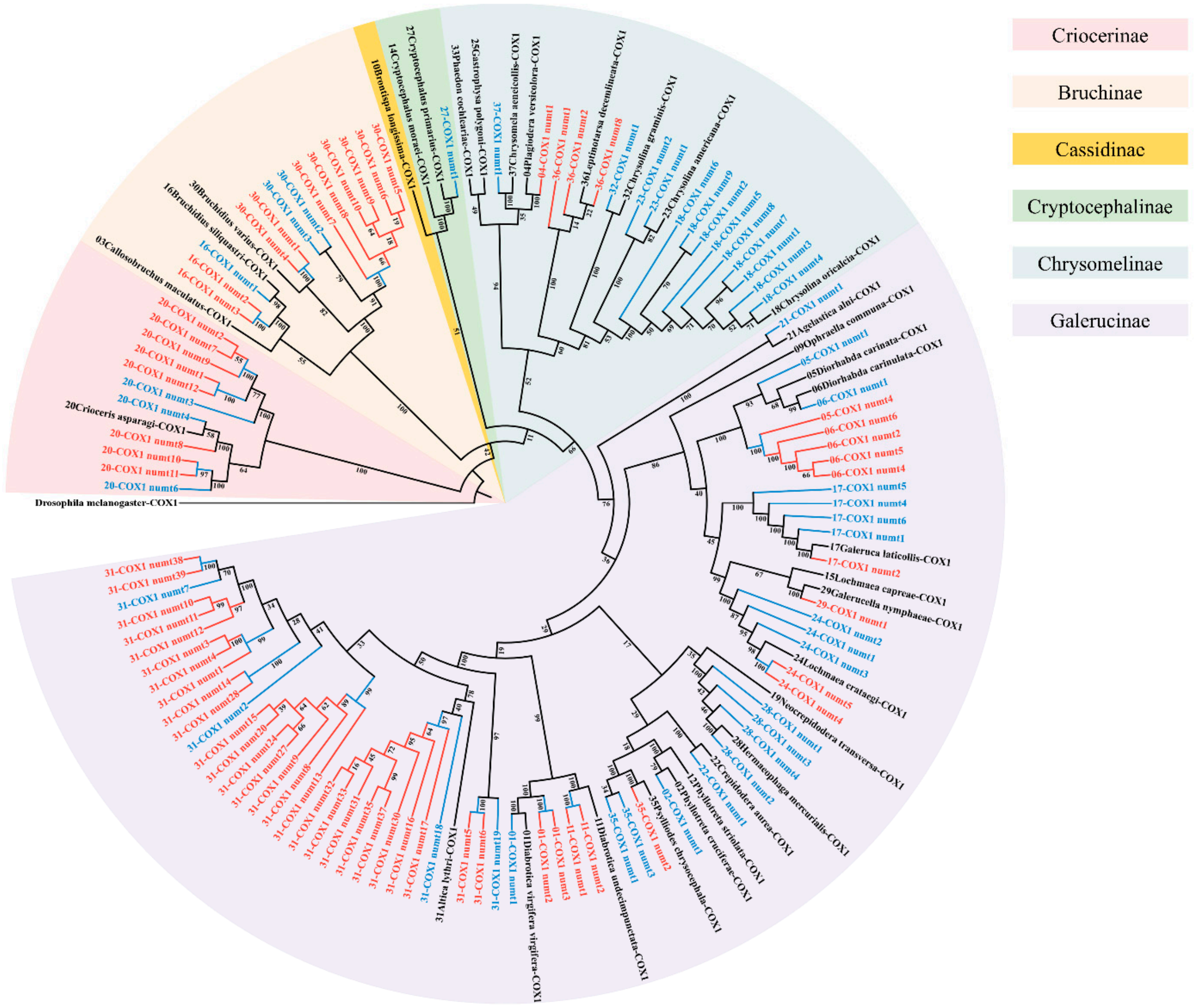

Figure S9: ML tree of the *COX1* and NUMT dataset without branch lengths.
