# Supplementary figures and images for: A Genome-Wide Analysis of Nuclear Mitochondrial DNA Sequences (NUMTs) in Chrysomelidae Species (Coleoptera)
Source: Insects. 2025 Feb 2;16(2):150. doi: 10.3390/insects16020150 (PMC11856540; doi:10.3390/insects16020150)

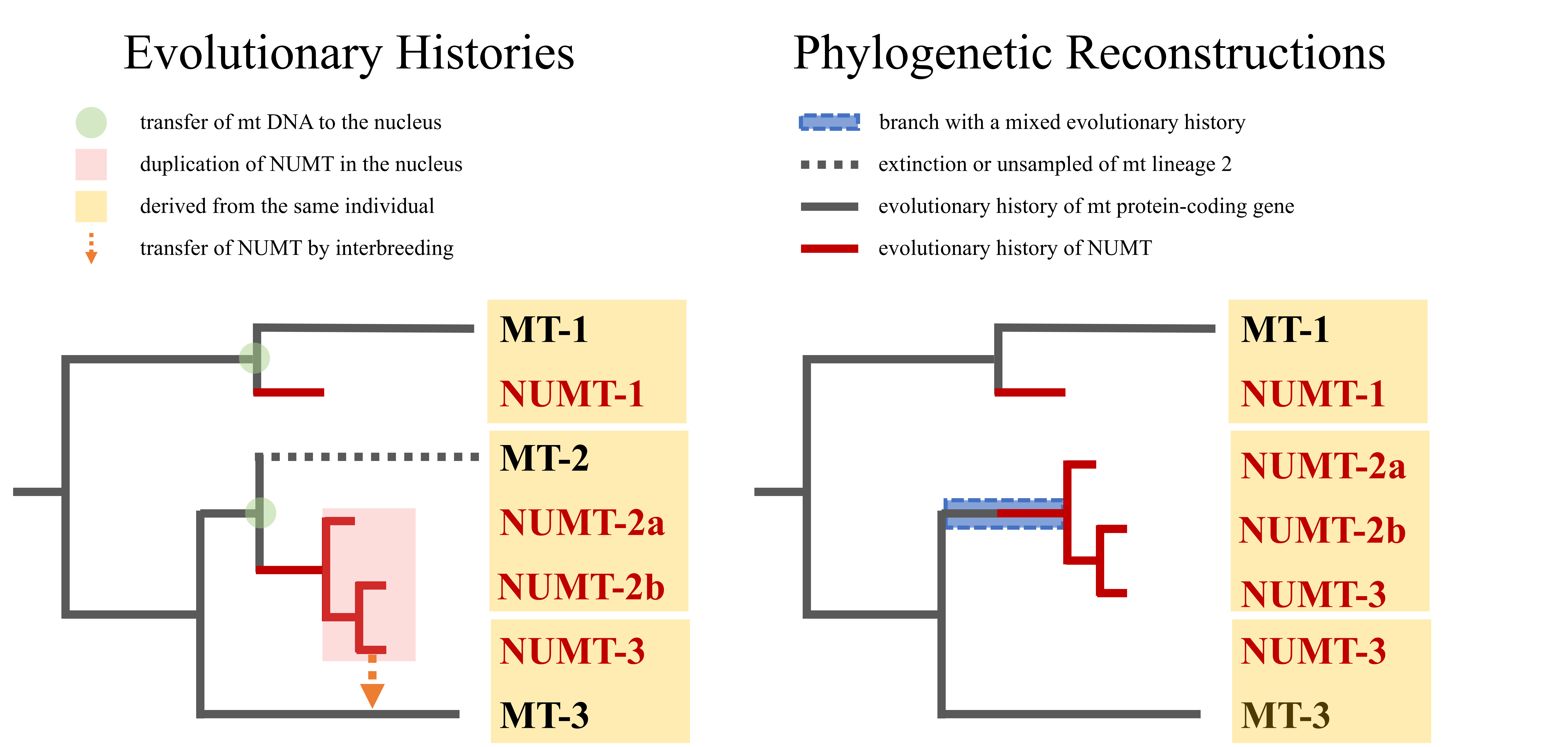

Supplement: Supplementary file 1 [file insects-16-00150-s001.zip › Figure S1.tif]

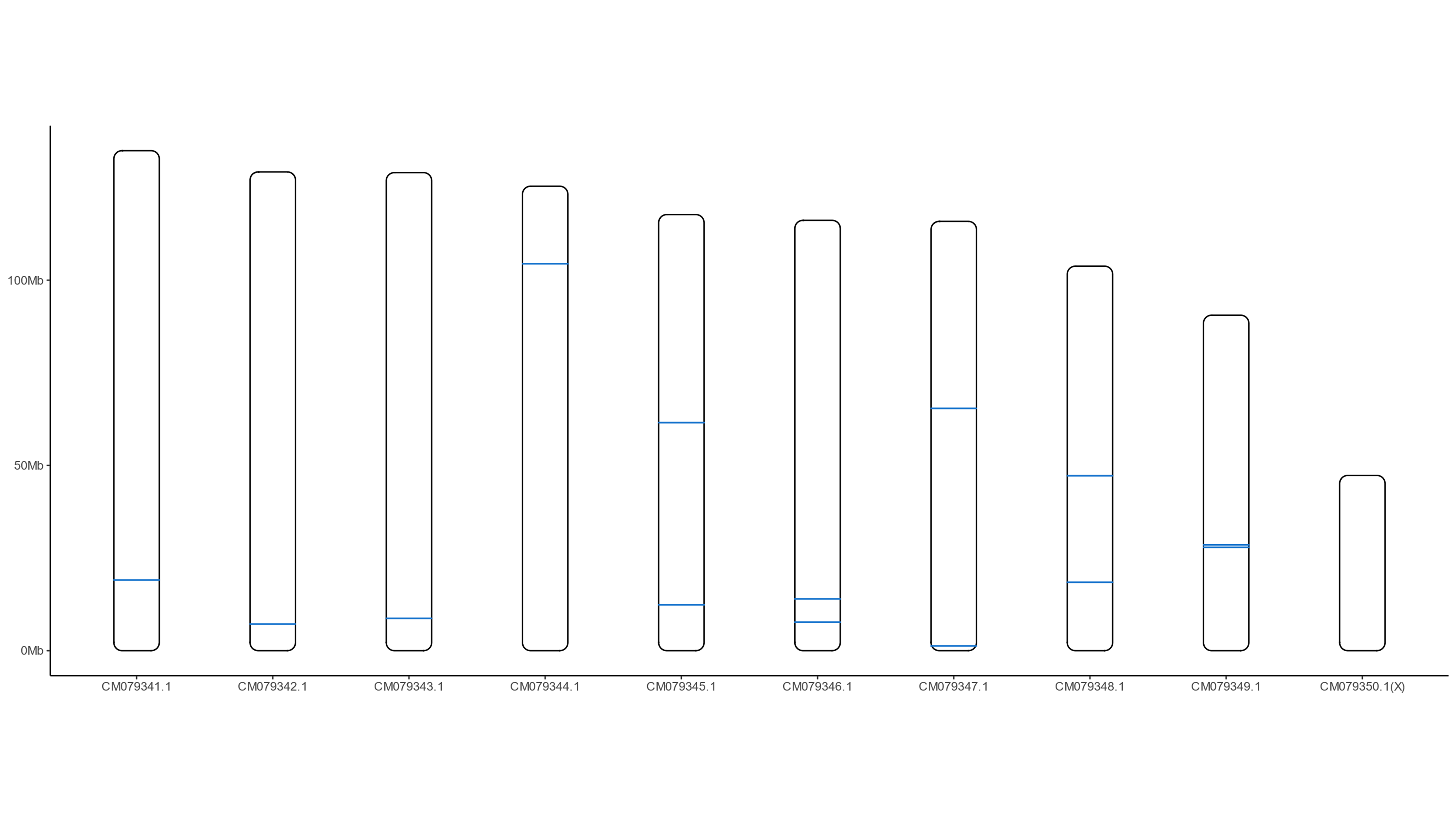

Supplement: Supplementary file 1 [file insects-16-00150-s001.zip › Figure S2a.tif]

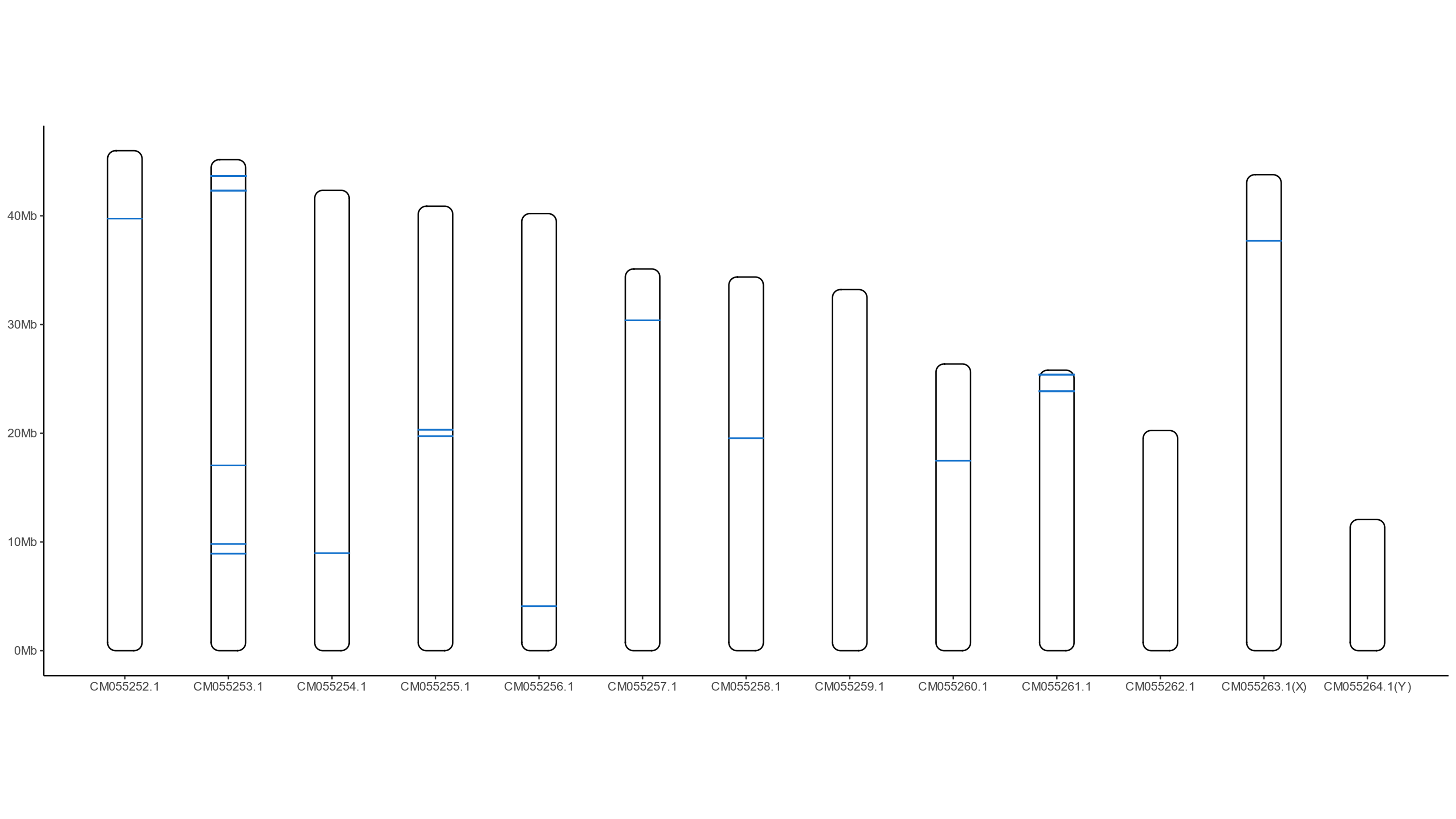

Supplement: Supplementary file 1 [file insects-16-00150-s001.zip › Figure S2b.tif]

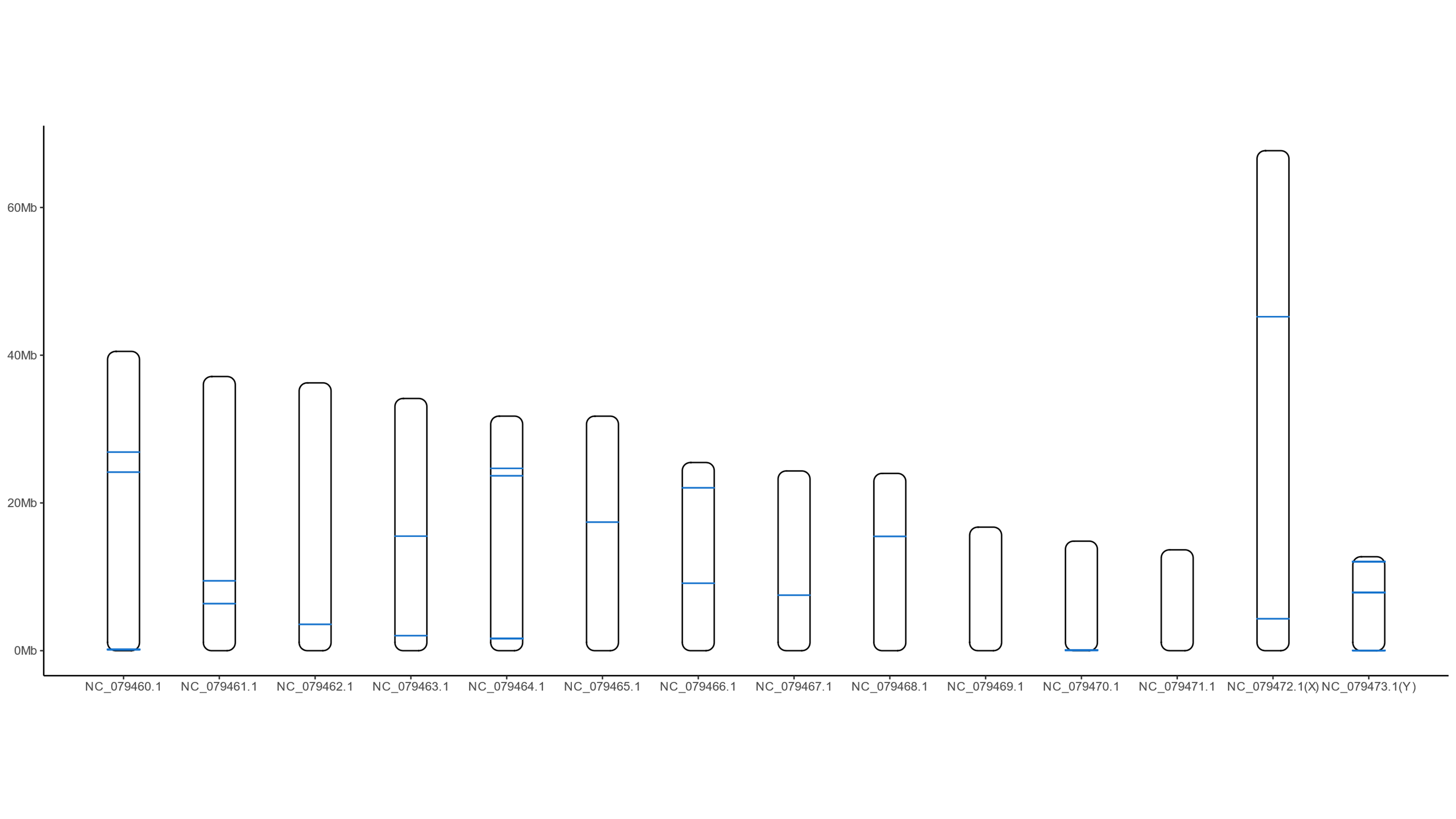

Supplement: Supplementary file 1 [file insects-16-00150-s001.zip › Figure S2c.tif]

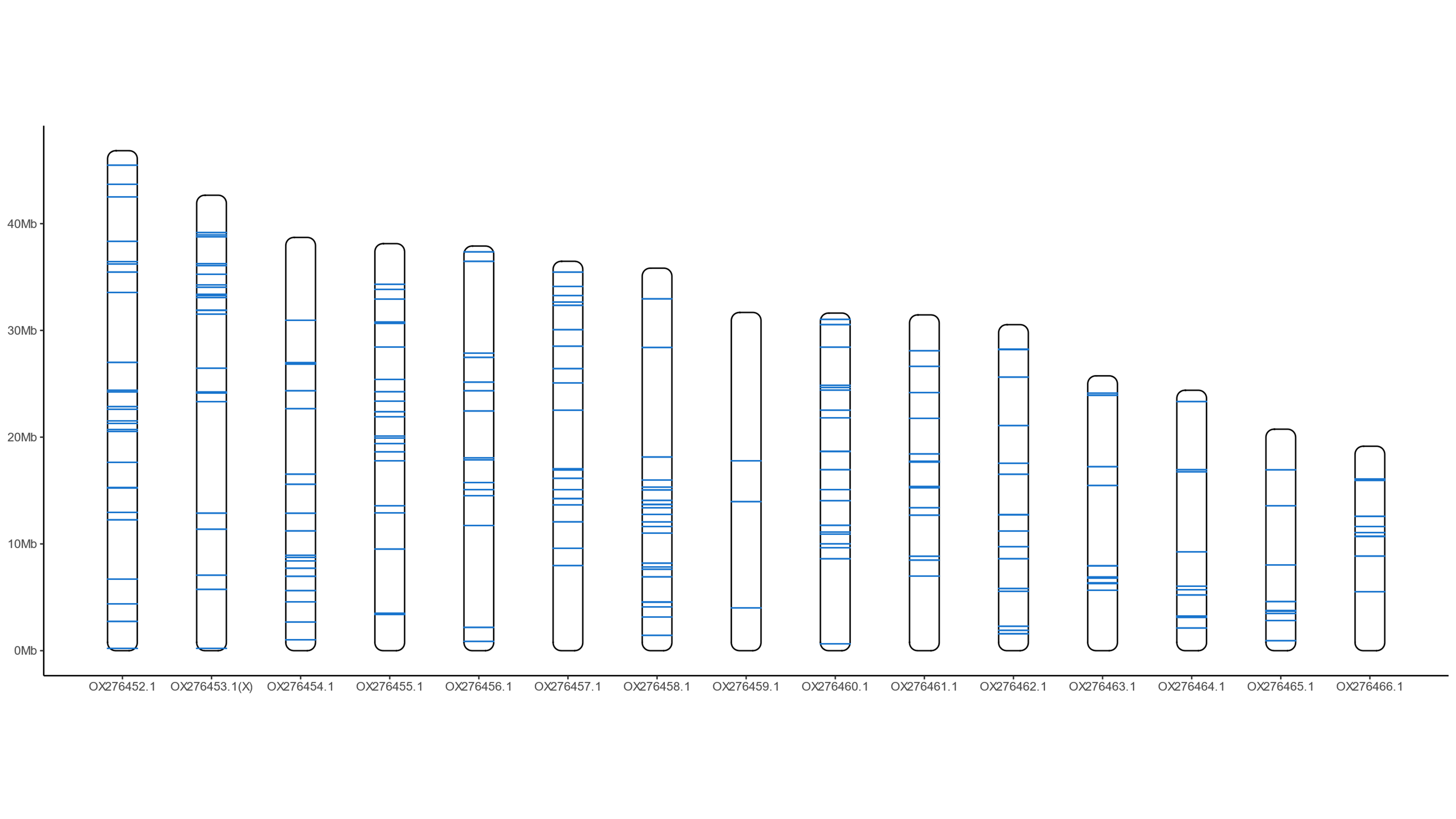

Supplement: Supplementary file 1 [file insects-16-00150-s001.zip › Figure S2d.tif]

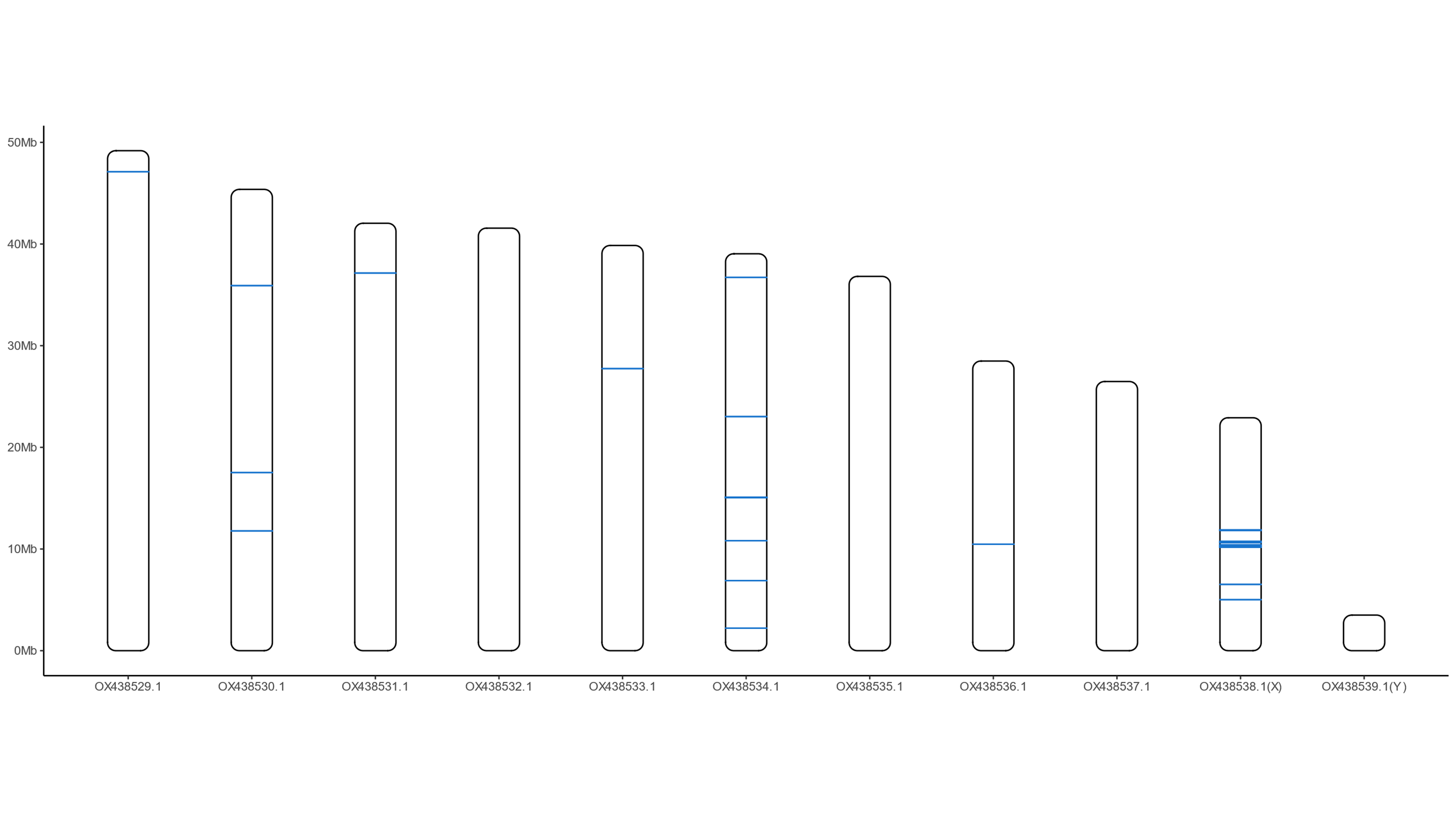

Supplement: Supplementary file 1 [file insects-16-00150-s001.zip › Figure S2e.tif]

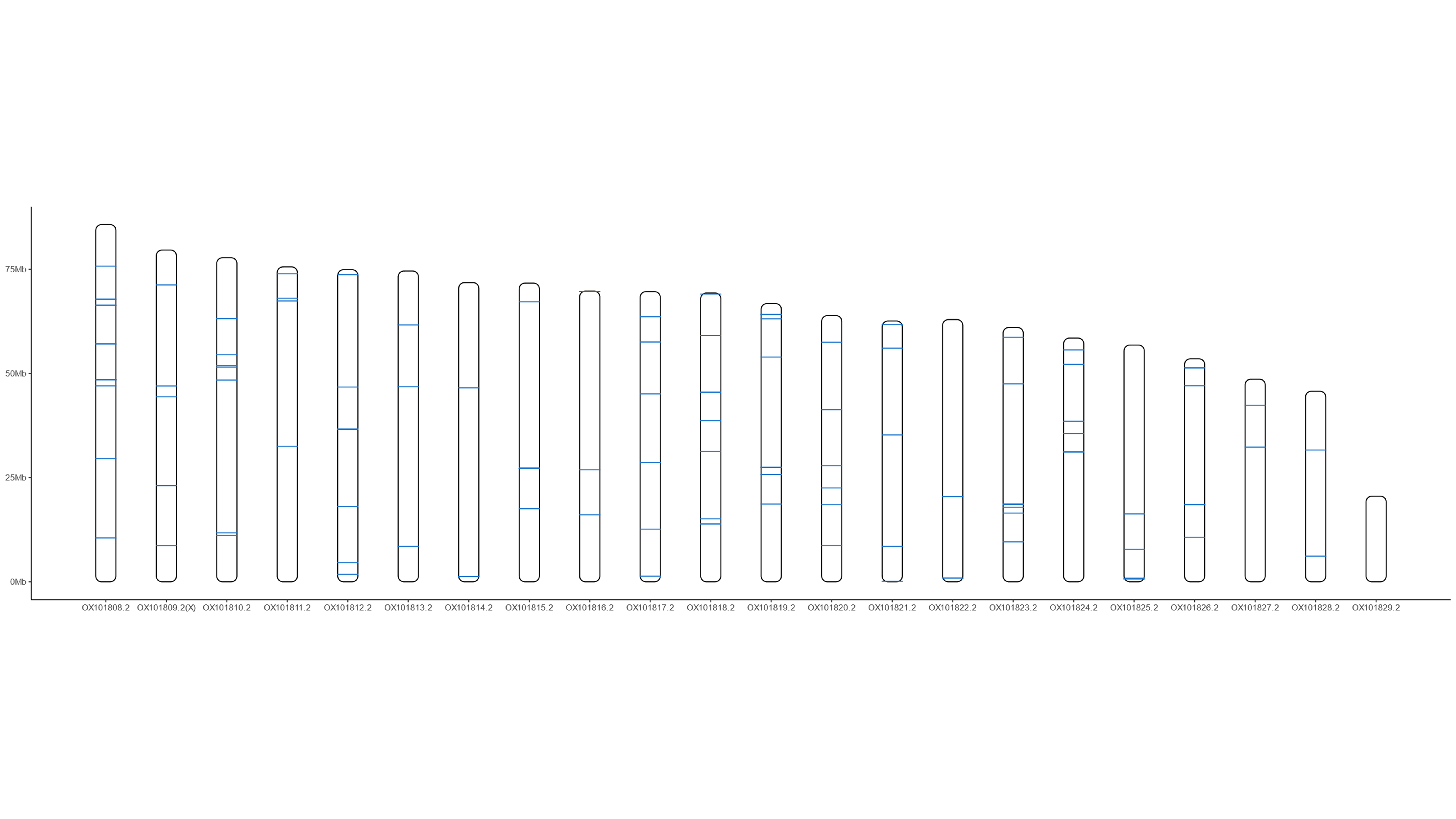

Supplement: Supplementary file 1 [file insects-16-00150-s001.zip › Figure S2f.tif]

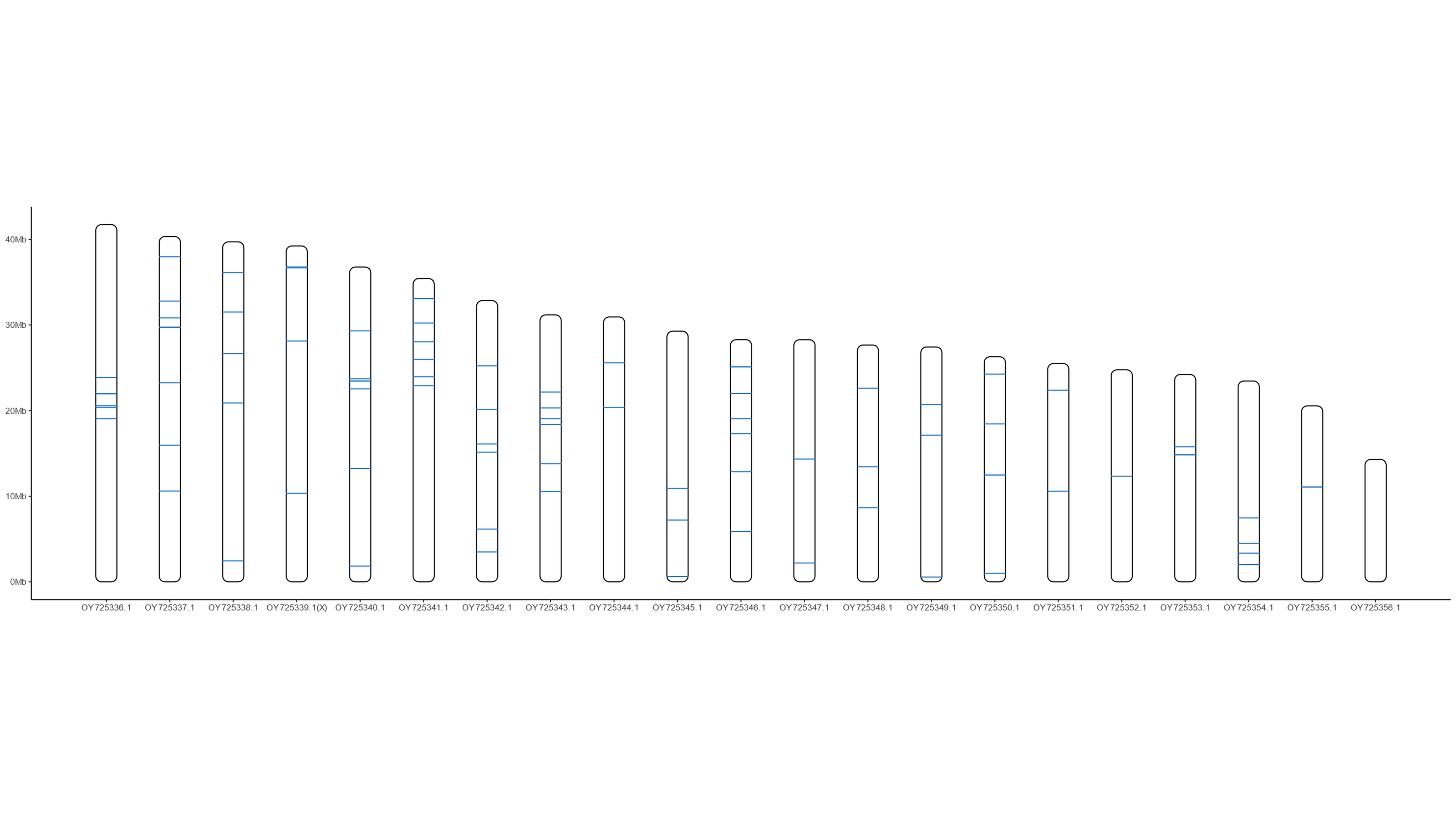

Supplement: Supplementary file 1 [file insects-16-00150-s001.zip › Figure S2g.tif]

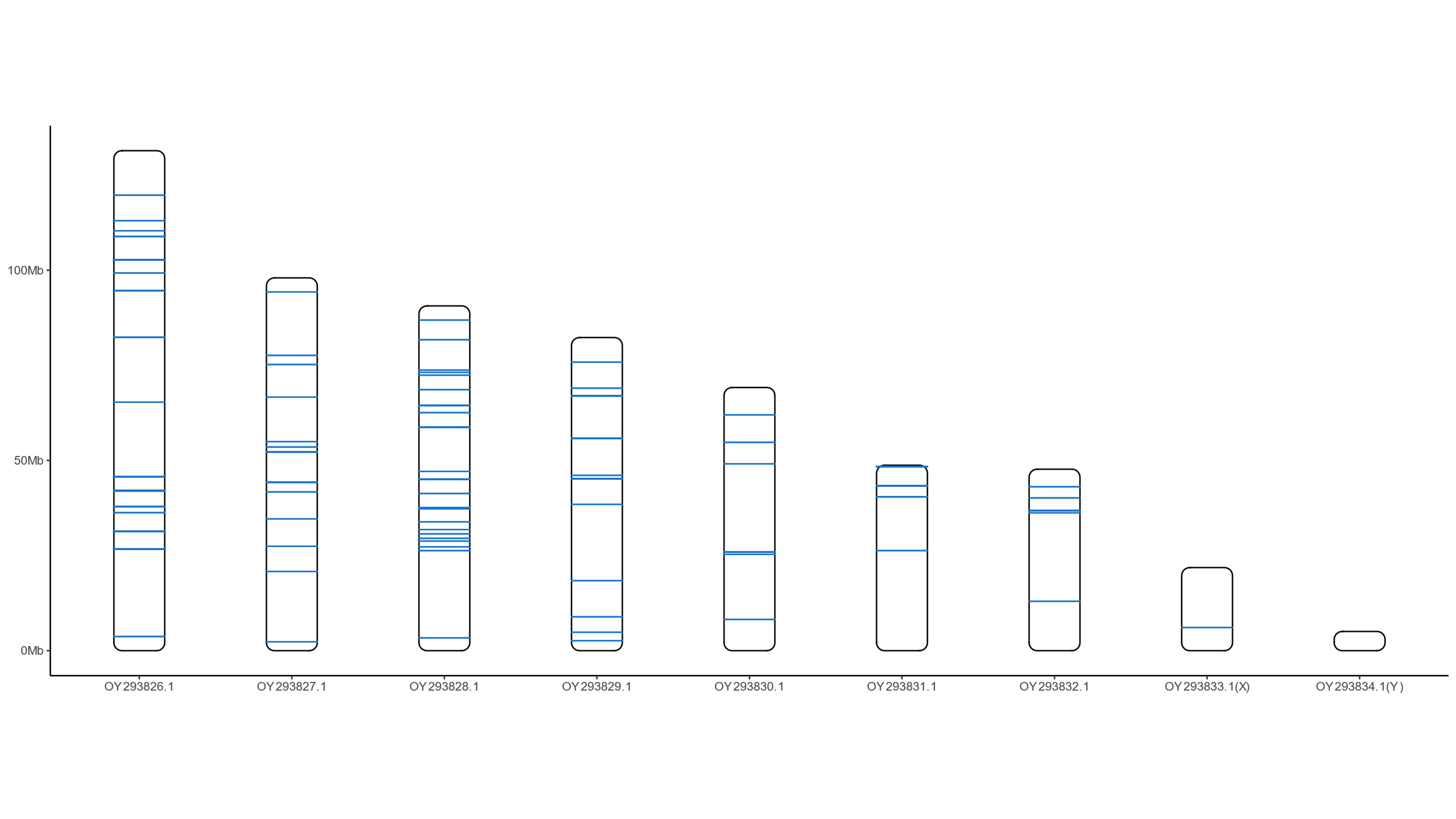

Supplement: Supplementary file 1 [file insects-16-00150-s001.zip › Figure S2h.tif]

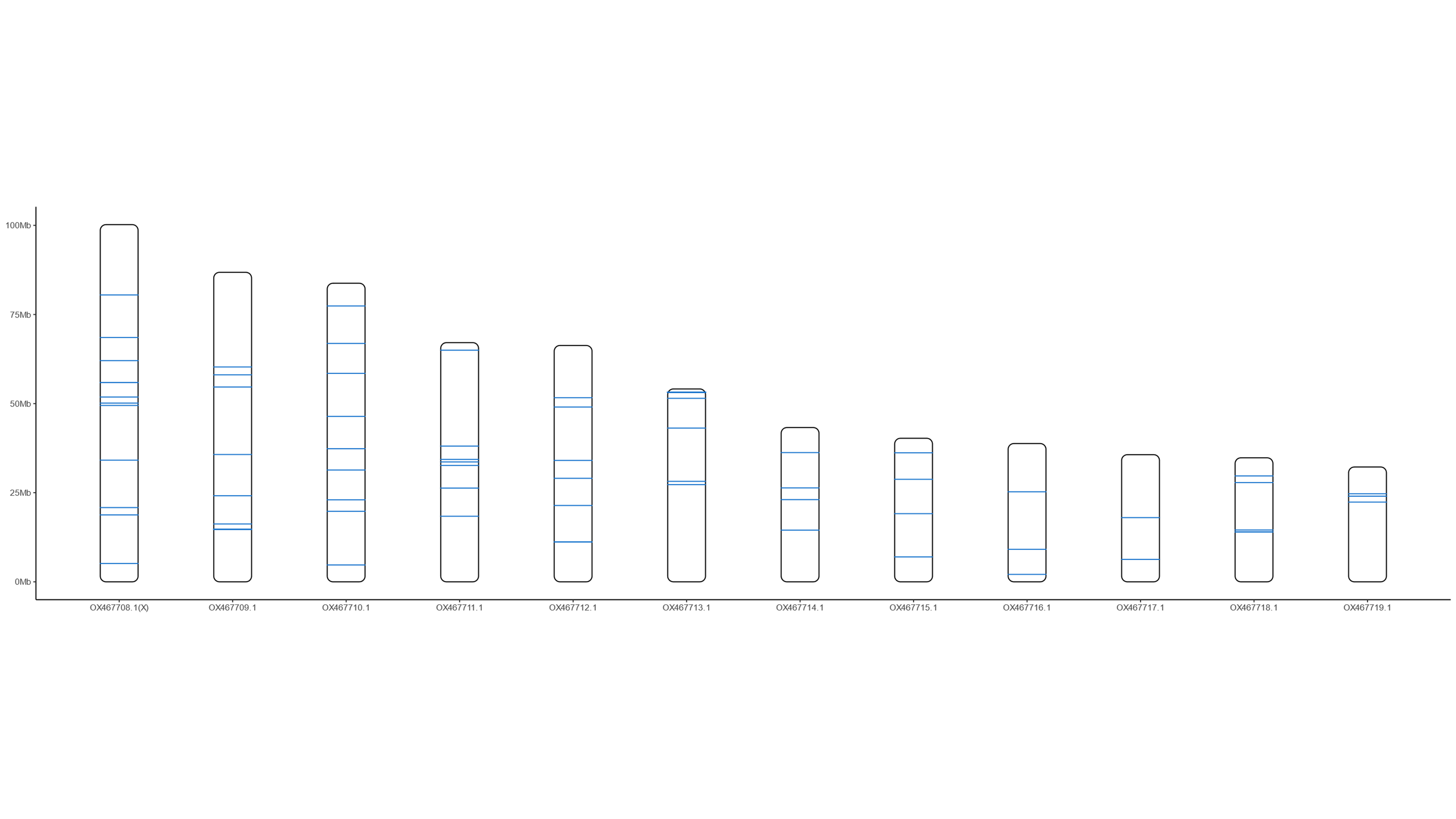

Supplement: Supplementary file 1 [file insects-16-00150-s001.zip › Figure S2i.tif]

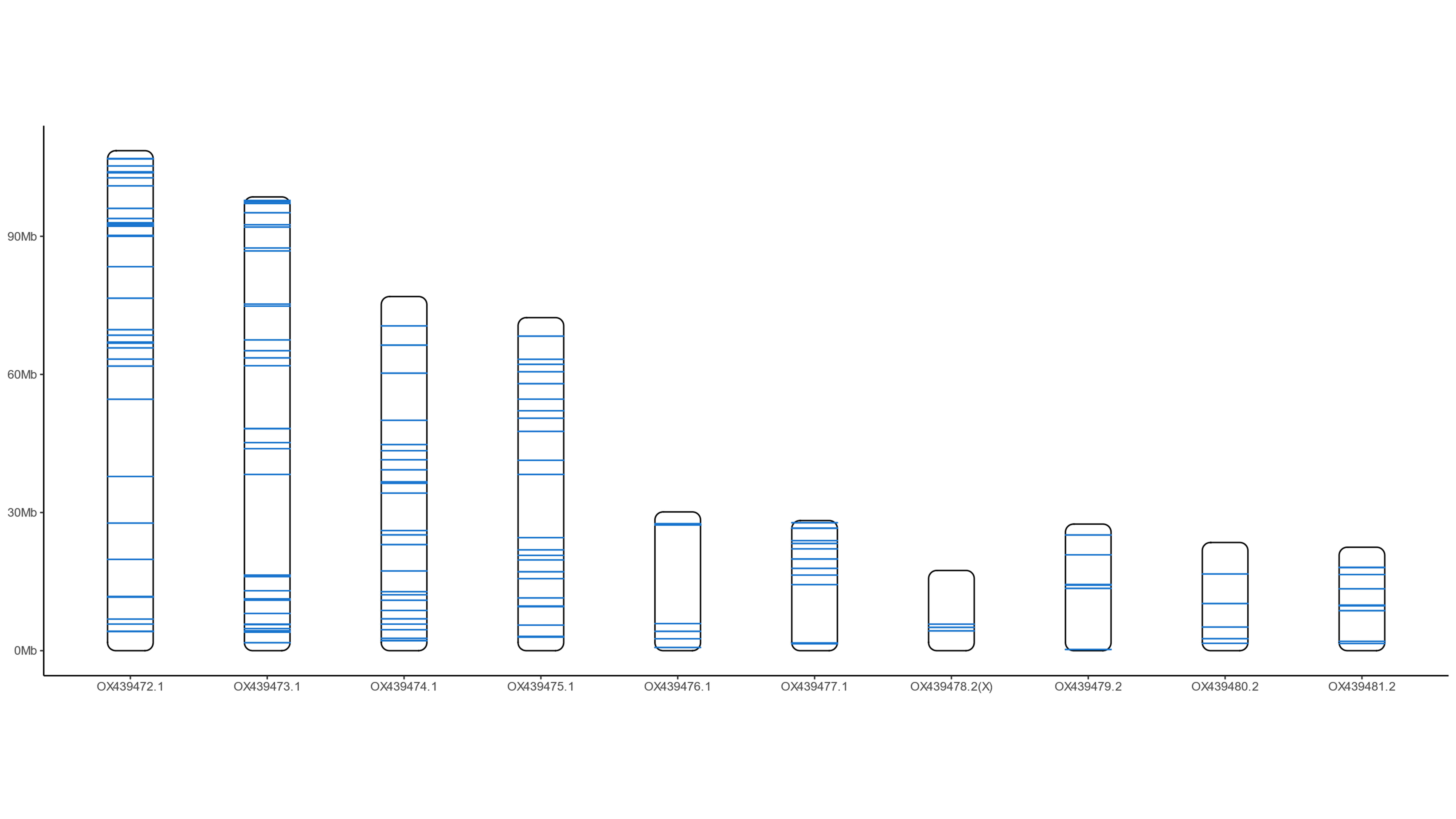

Supplement: Supplementary file 1 [file insects-16-00150-s001.zip › Figure S2j.tif]

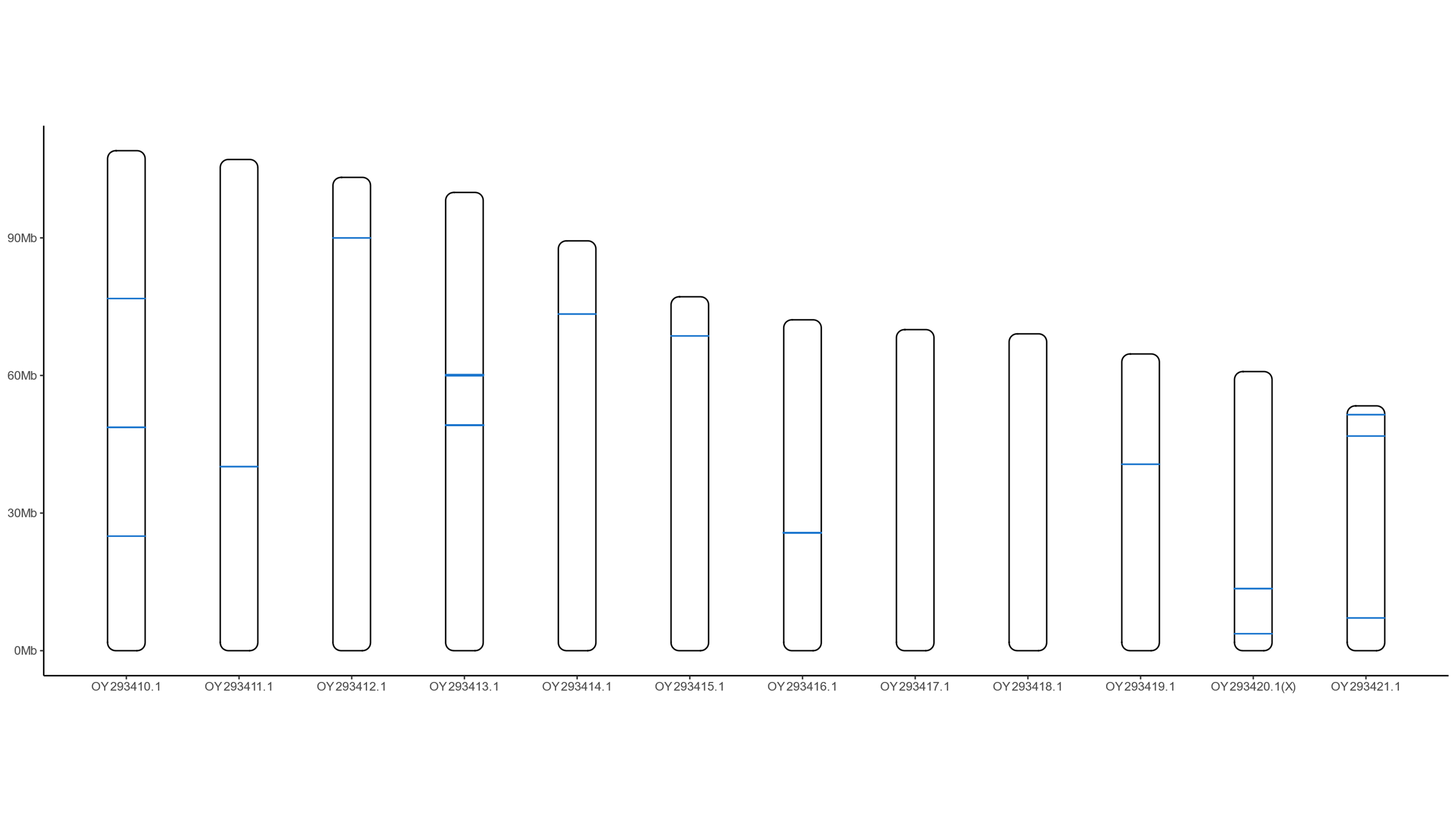

Supplement: Supplementary file 1 [file insects-16-00150-s001.zip › Figure S2k.tif]

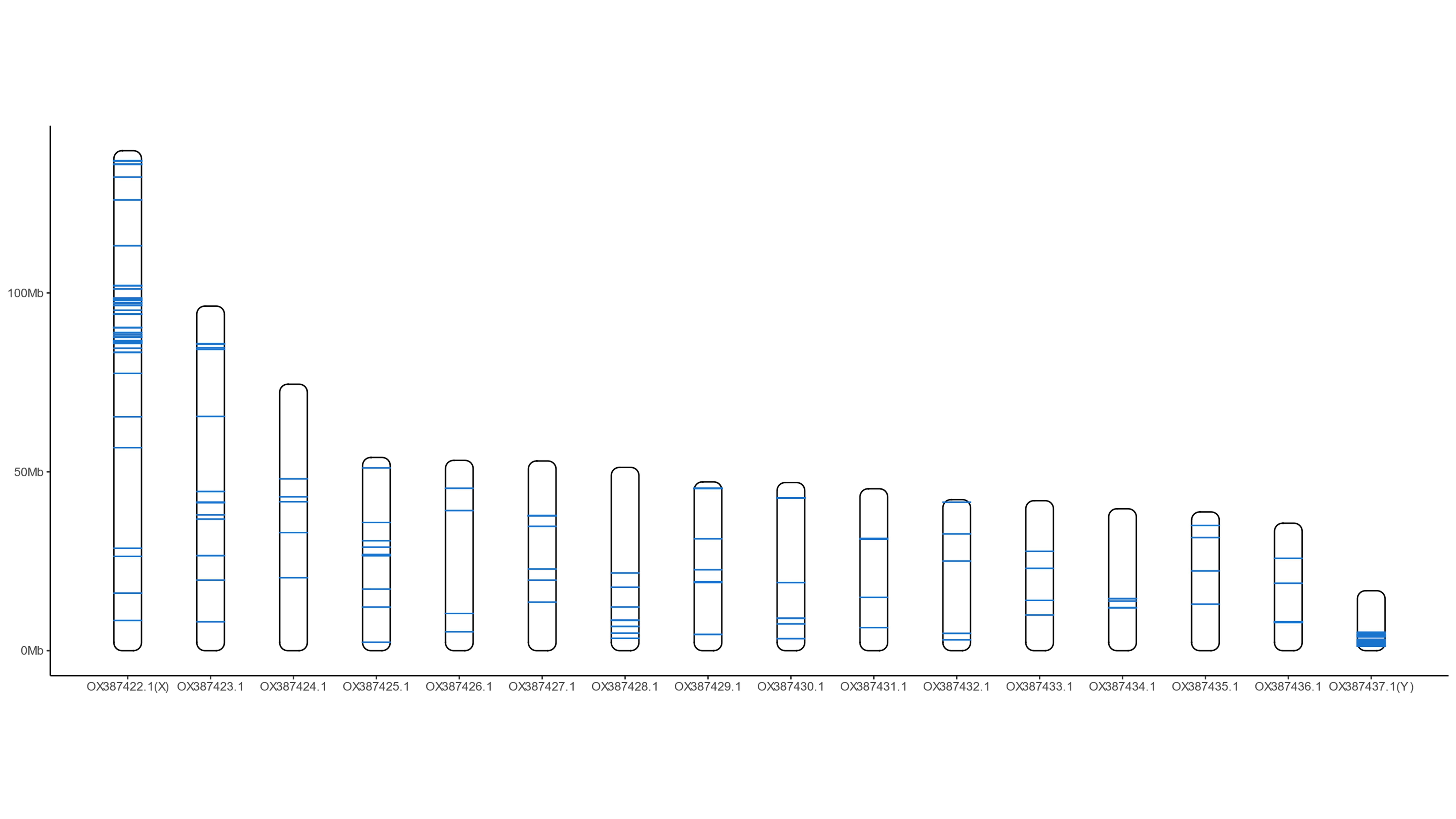

Supplement: Supplementary file 1 [file insects-16-00150-s001.zip › Figure S2l.tif]

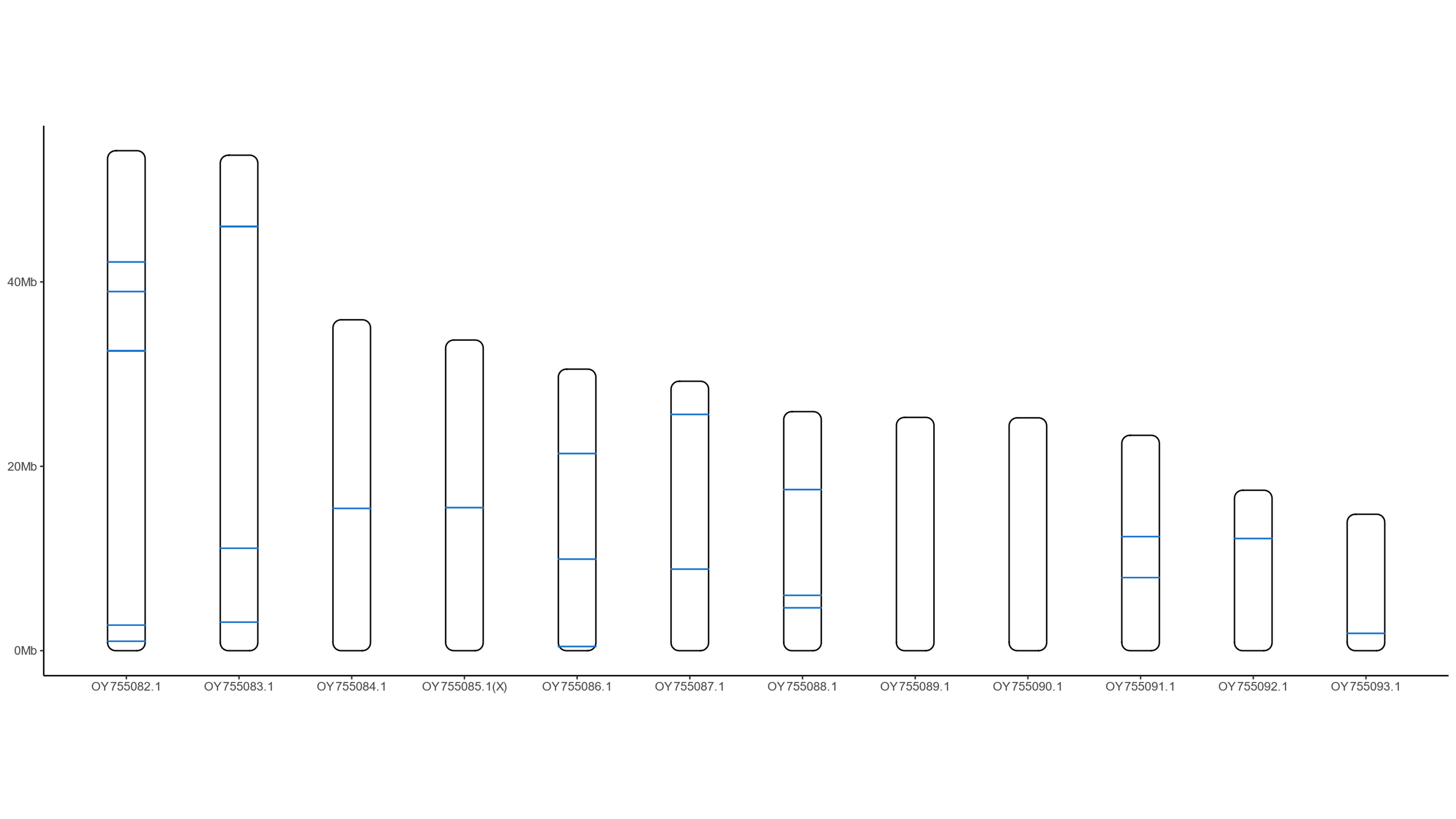

Supplement: Supplementary file 1 [file insects-16-00150-s001.zip › Figure S2m.tif]

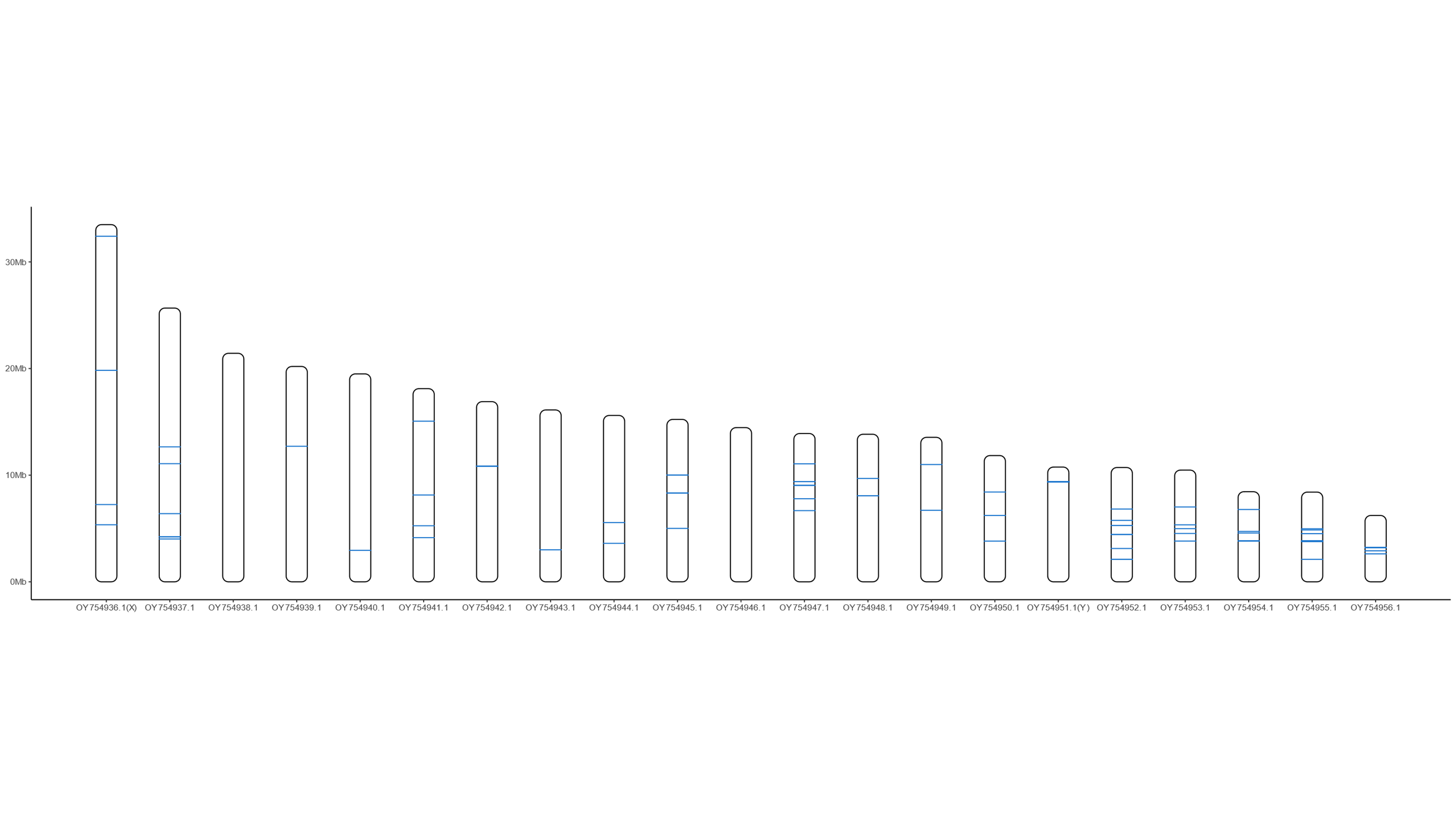

Supplement: Supplementary file 1 [file insects-16-00150-s001.zip › Figure S2n.tif]

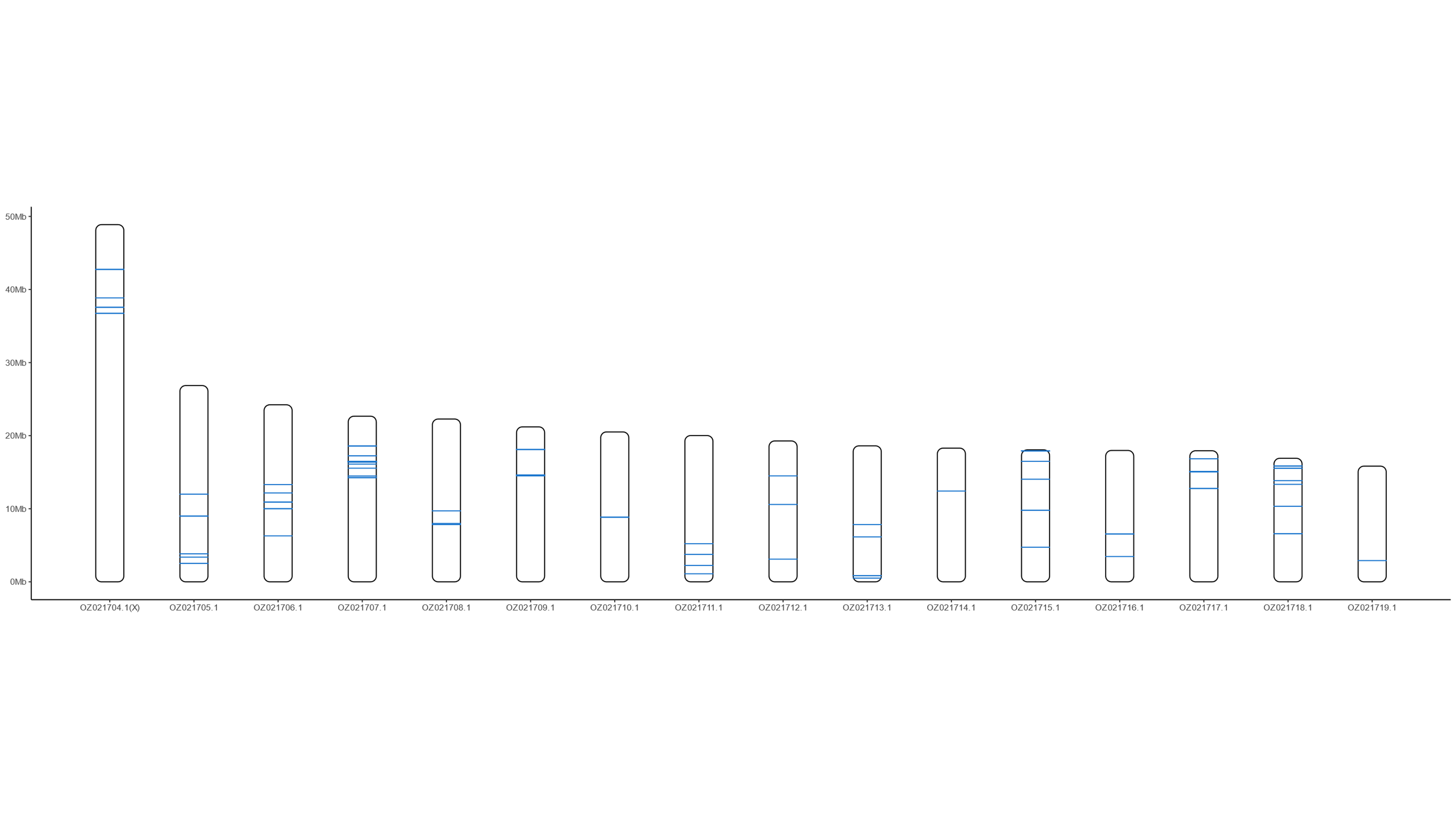

Supplement: Supplementary file 1 [file insects-16-00150-s001.zip › Figure S2o.tif]

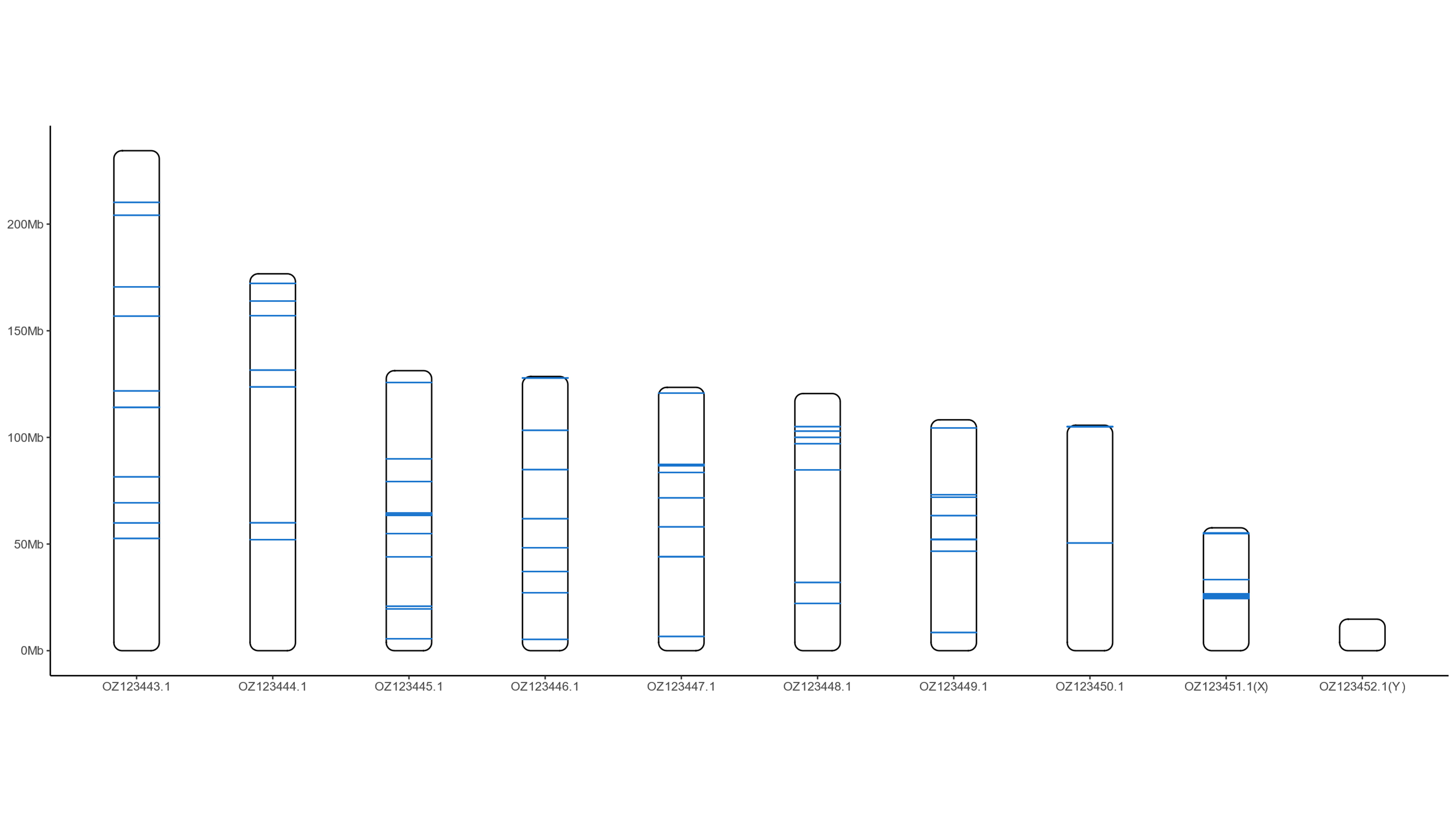

Supplement: Supplementary file 1 [file insects-16-00150-s001.zip › Figure S2p.tif]

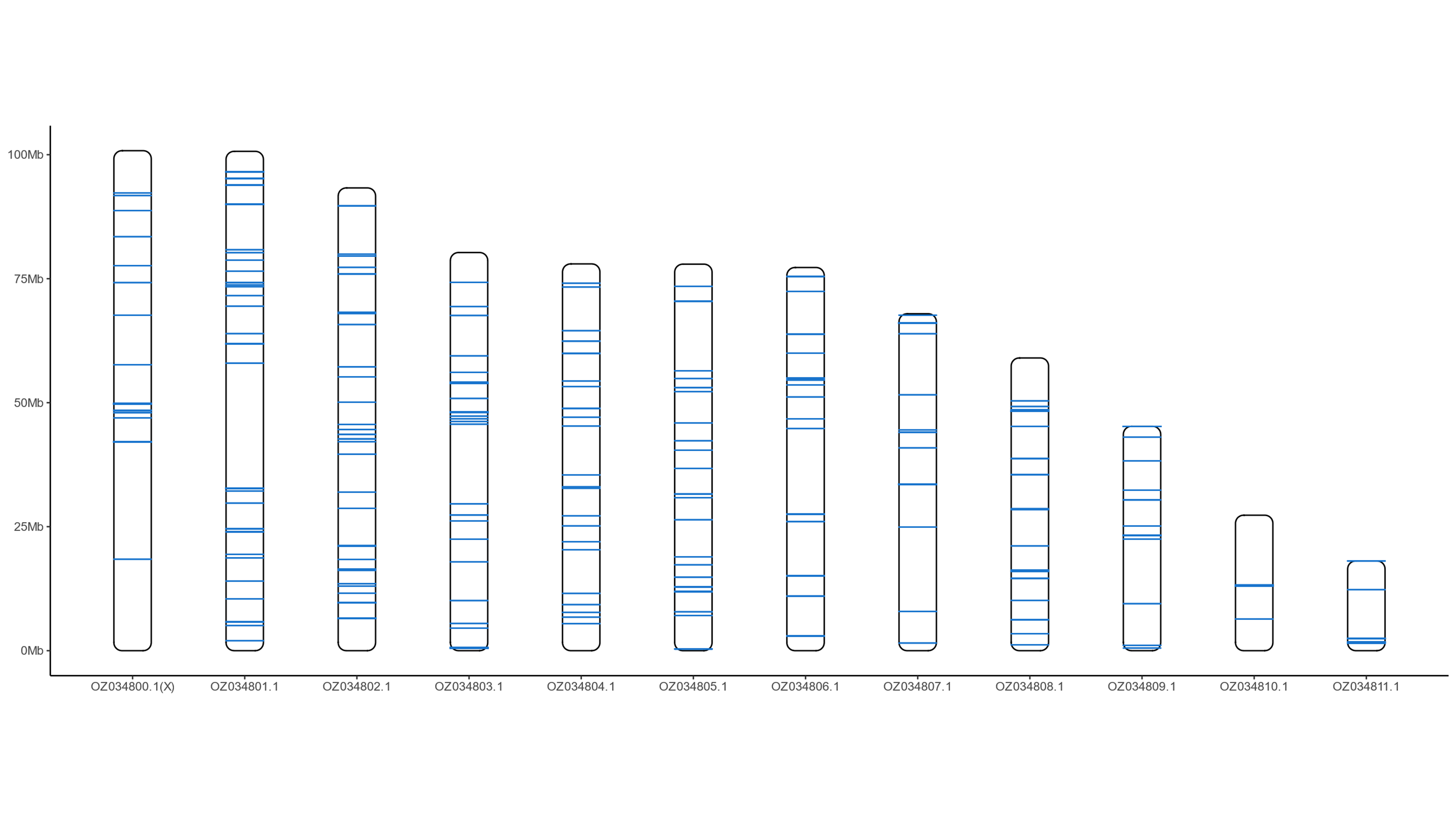

Supplement: Supplementary file 1 [file insects-16-00150-s001.zip › Figure S2q.tif]

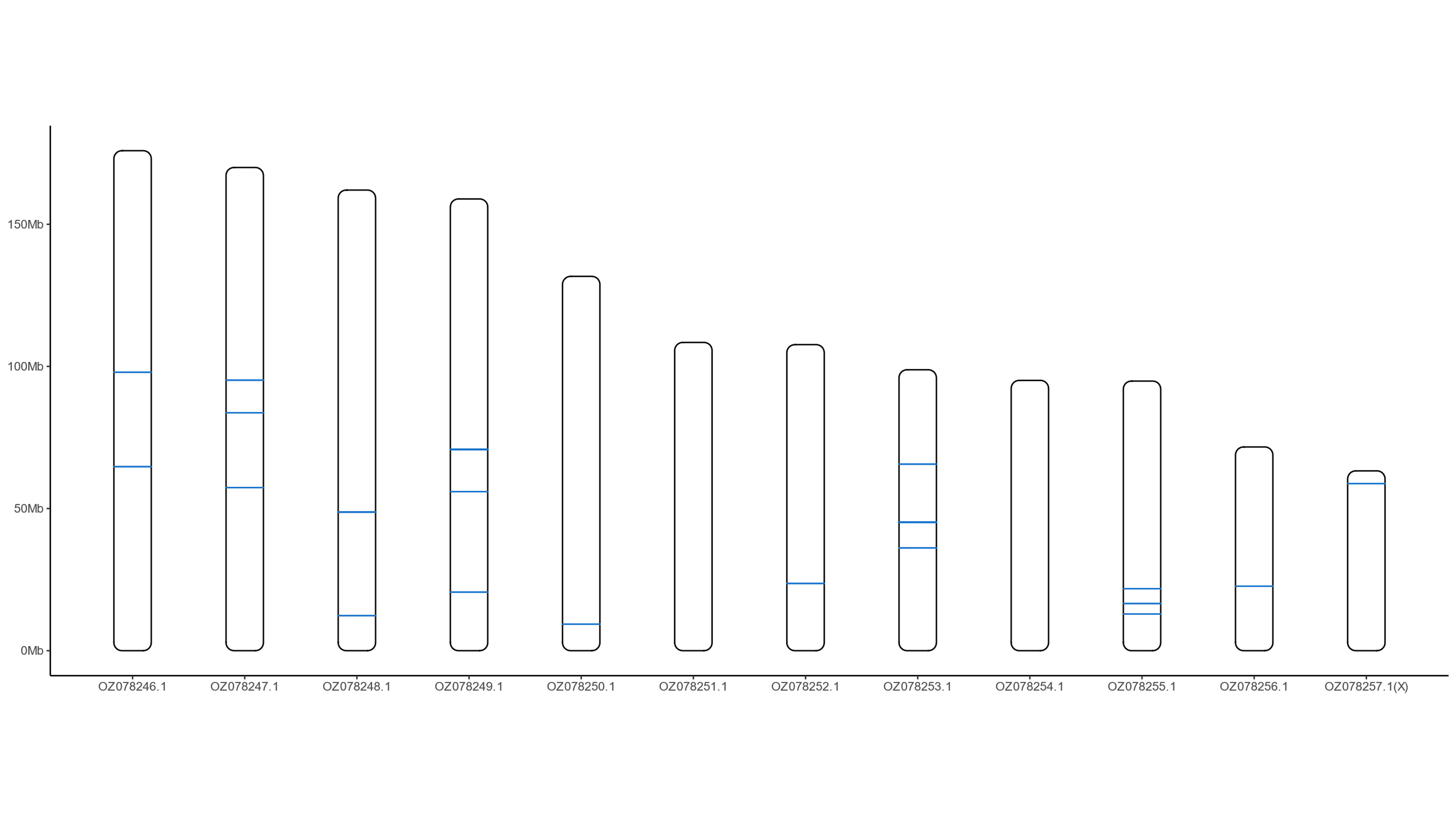

Supplement: Supplementary file 1 [file insects-16-00150-s001.zip › Figure S2r.tif]

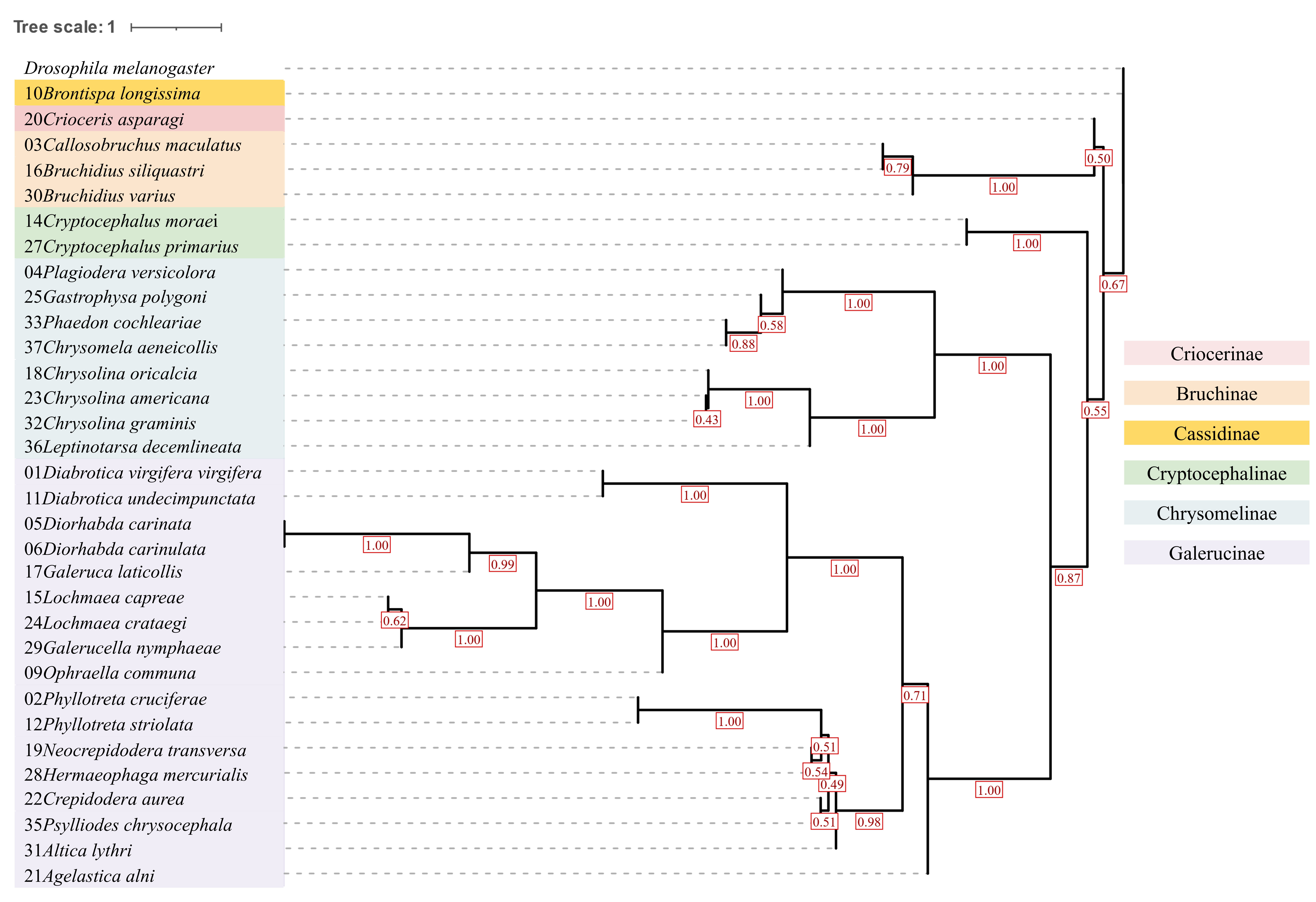

Supplement: Supplementary file 1 [file insects-16-00150-s001.zip › Figure S4.tif]

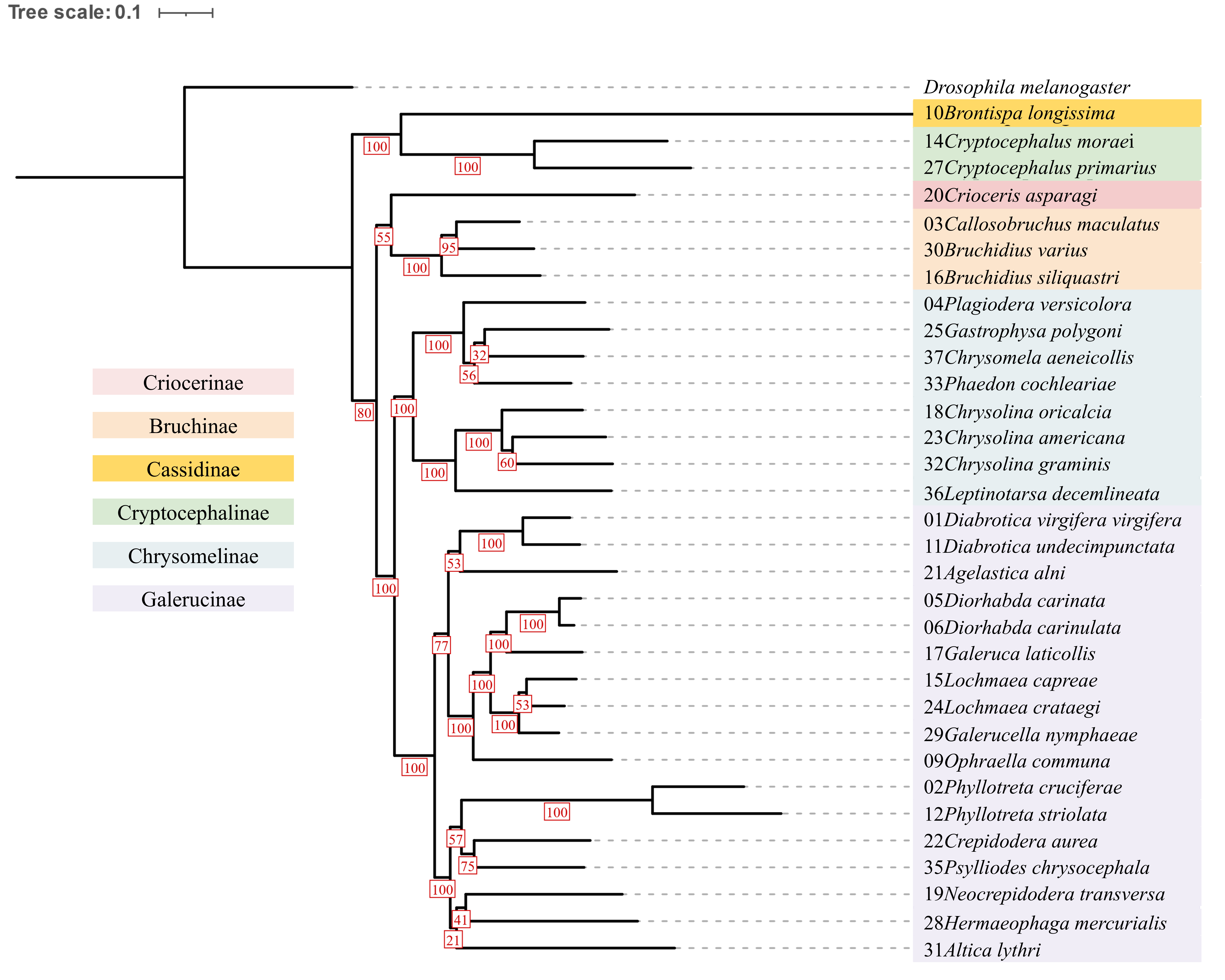

Supplement: Supplementary file 1 [file insects-16-00150-s001.zip › Figure S5a.tif]

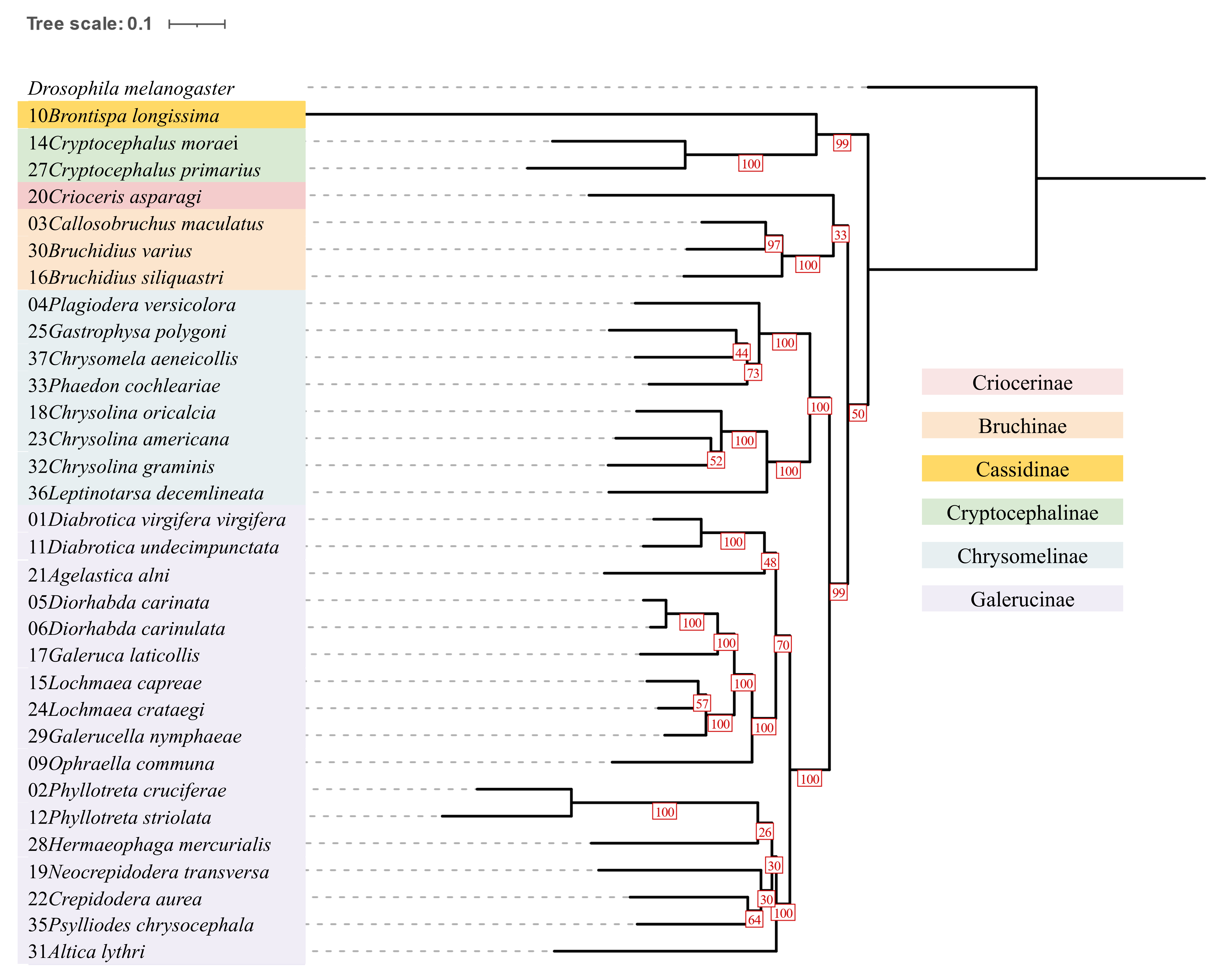

Supplement: Supplementary file 1 [file insects-16-00150-s001.zip › Figure S5b.tif]

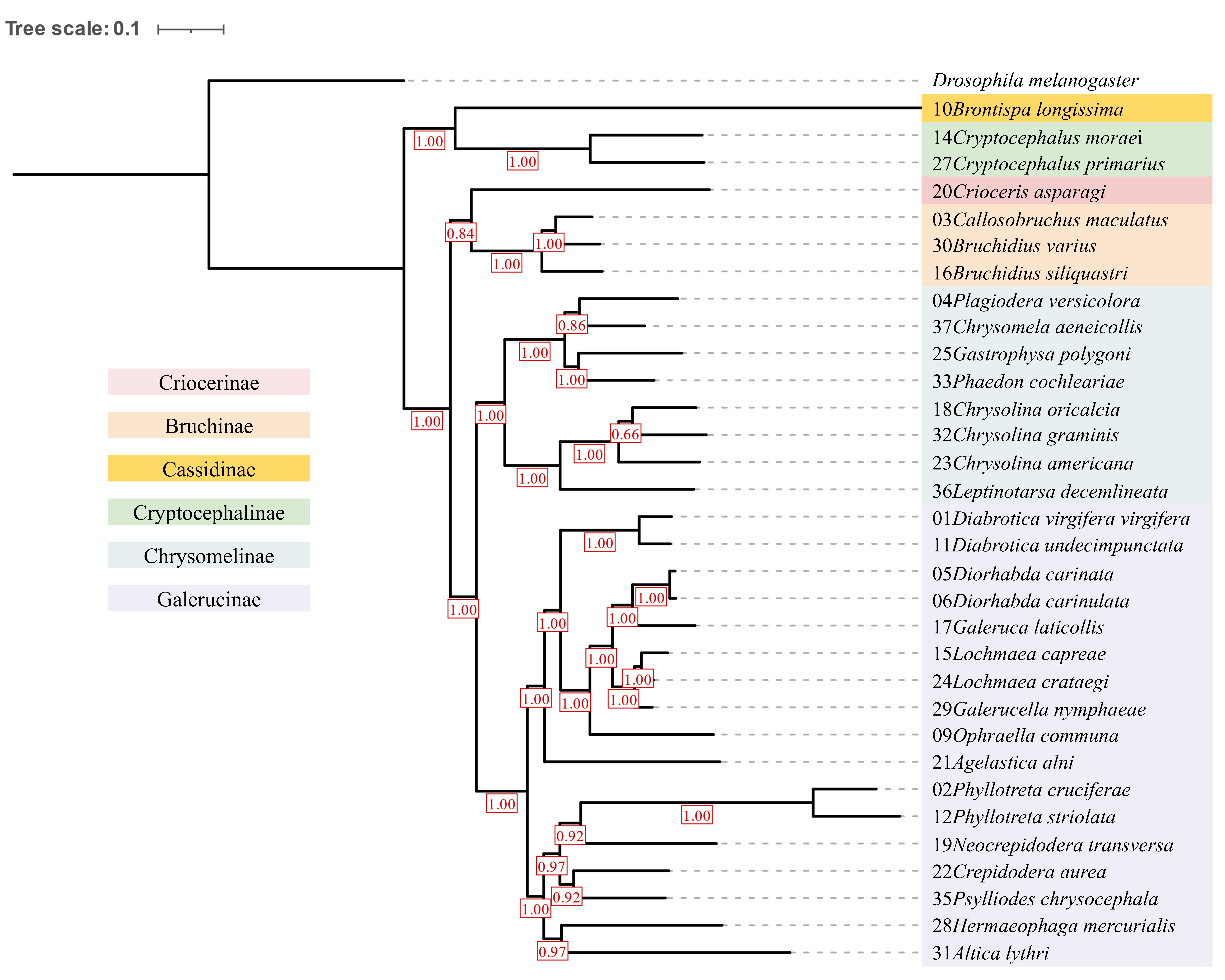

Supplement: Supplementary file 1 [file insects-16-00150-s001.zip › Figure S6.tif]

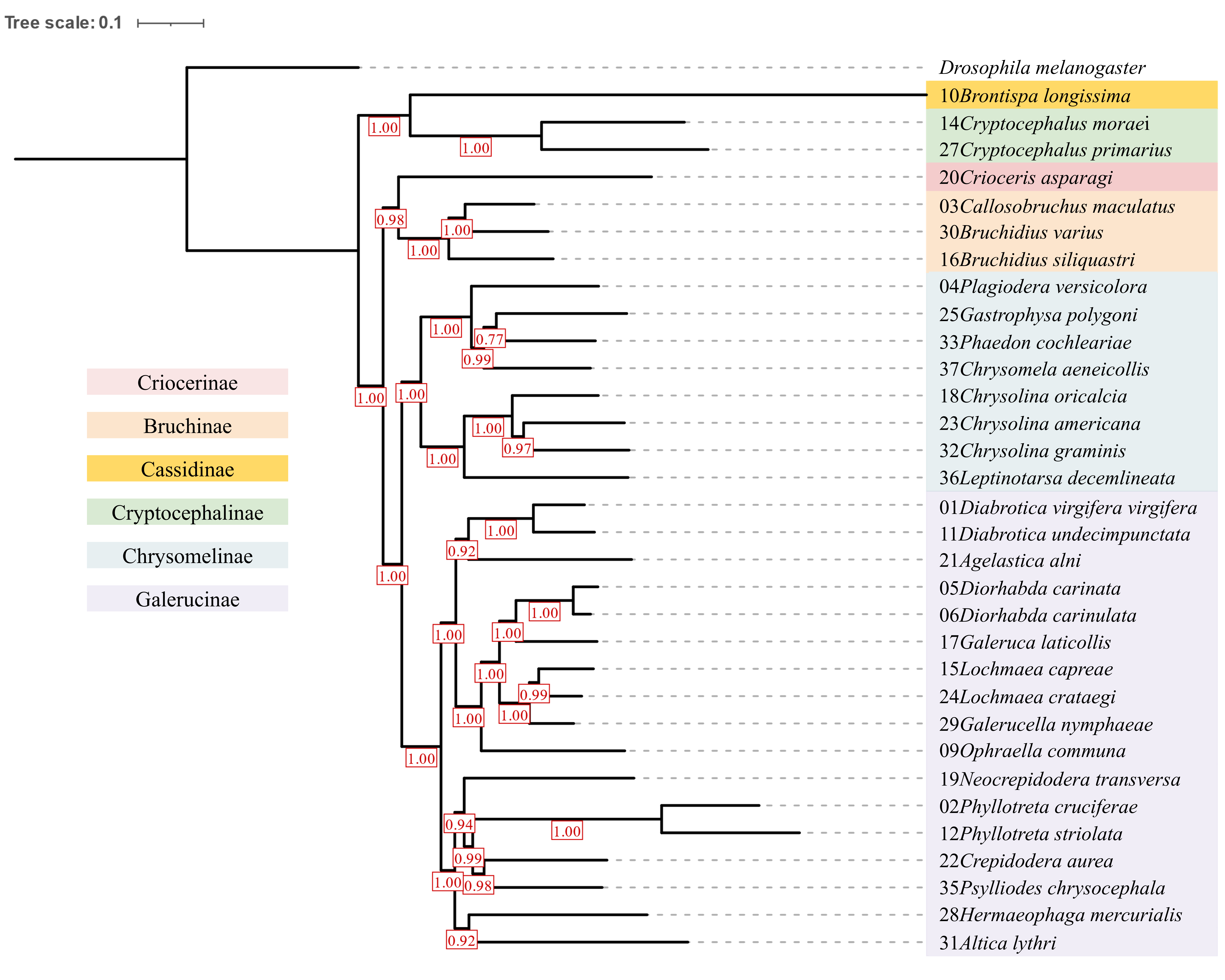

Supplement: Supplementary file 1 [file insects-16-00150-s001.zip › Figure S7a.tif]

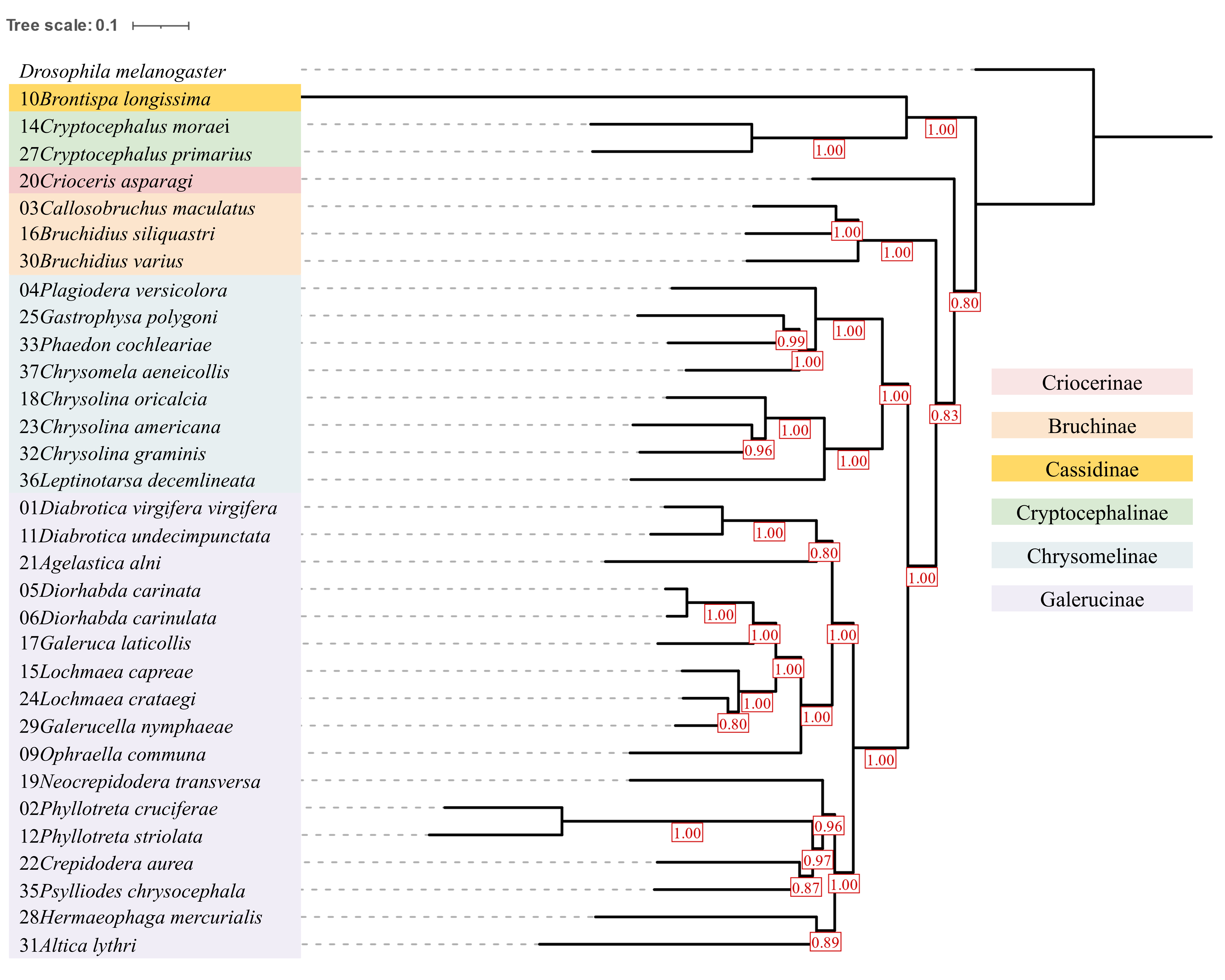

Supplement: Supplementary file 1 [file insects-16-00150-s001.zip › Figure S7b.tif]

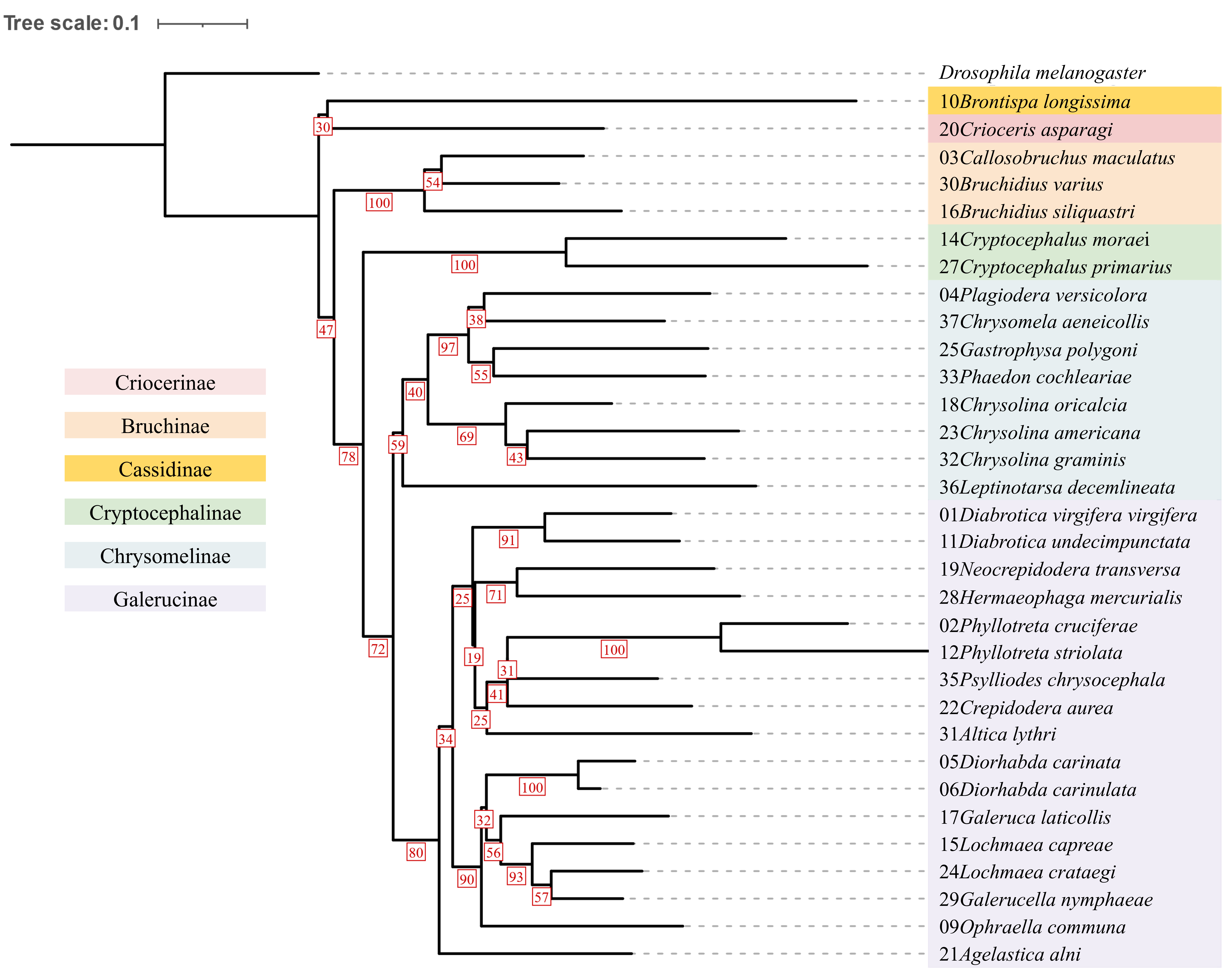

Supplement: Supplementary file 1 [file insects-16-00150-s001.zip › Figure S8.tif]

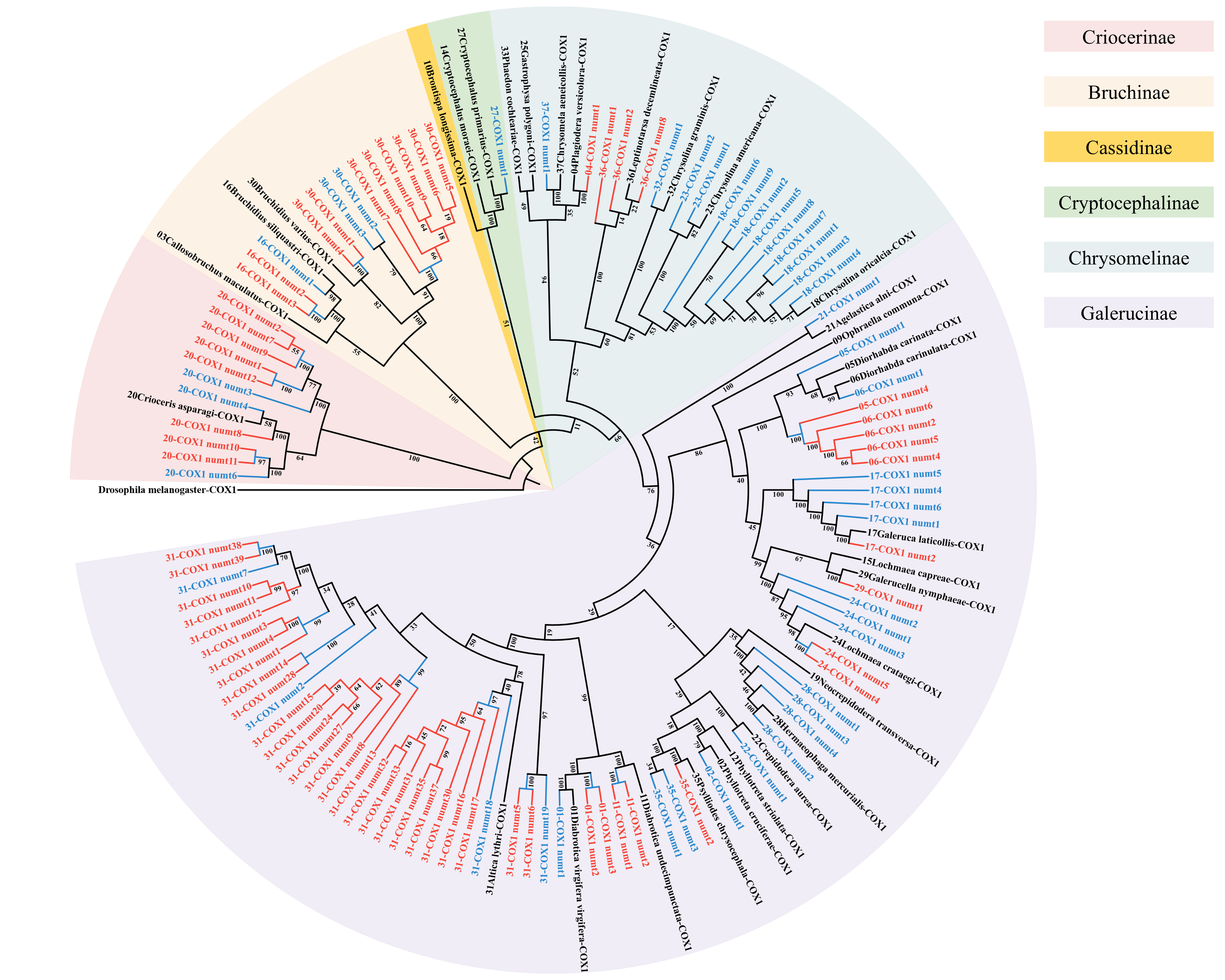

Supplement: Supplementary file 1 [file insects-16-00150-s001.zip › Figure S9.tif]
